# Inflammation-driven bone erosion and reactive remodeling in experimental arthritis revealed by cortical bone surface analysis

**DOI:** 10.1186/s13075-026-03874-y

**Published:** 2026-08-06

**Authors:** Anders Nguyen, David A. McBride, Miriam Bollmann, Liam Davidsson, Nour Dada, Taymaa Sami, Lisi Wagener, Carmen Corciulo, Muhammad Arif, Linda C. Johansson, Mattias N. D. Svensson

**Affiliations:** 1 Department of Rheumatology and Inflammation Research, Sahlgrenska Academy, Institute of Medicine, University of Gothenburg, Gothenburg, Sweden; 2 Calibr-Skaggs Institute for Innovative Medicines, The Scripps Research Institute, La Jolla, CA USA; 3 Department of Molecular and Clinical Medicine, Sahlgrenska Academy, Institute of Medicine, University of Gothenburg, Gothenburg, Sweden; 4 Department of Pharmacology, Sahlgrenska Academy, Institute of Neuroscience and Physiology, University of Gothenburg, Gothenburg, Sweden; 5 SciLifeLab, University of Gothenburg, Gothenburg, Sweden; 6 Department of Medical Biochemistry and Cell biology, Sahlgrenska Academy, Institute of Biomedicin, University of Gothenburg, Gothenburg, Sweden

**Keywords:** Micro-computed tomography, Rheumatoid arthritis, Cortical bone erosions, Bone remodeling, RANKL

## Abstract

**Background:**

Pre-clinical models of inflammatory arthritis are widely used to investigate mechanisms of disease pathology and evaluate therapeutic interventions. However, the sequence of inflammatory activation, bone erosion and reactive bone remodeling remains incompletely defined. We therefore sought to define the time-dependent relationship between inflammation and structural bone remodeling in experimental arthritis.

**Methods:**

K/BxN serum-transfer–induced arthritis was induced in male C57BL/6J mice, with longitudinal assessment of clinical disease, histopathology, joint gene expression and structural bone changes during disease progression. µCT analyses included semi-quantitative scoring, conventional morphometric parameters and BRAVO, our in-house µCT-based cortical surface roughness analysis workflow. Anti-RANKL treatment was used to evaluate therapeutic modulation of structural outcomes.

**Results:**

Synovial inflammation preceded histologically detectable cartilage erosion and bone erosion. Transcriptional activation of inflammatory and osteoclastogenic genes defined a pre-erosive molecular phase preceding measurable structural damage, whereas osteoblast- and matrix remodeling–associated genes increased during the reparative phase. Conventional µCT morphometrics and BRAVO both captured the overall erosive trajectory. However, BRAVO demonstrated improved discrimination of disease-associated cortical surface changes together with greater robustness, reduced age dependency, and clearer resolution of erosive and remodeling phases across disease progression. Anti-RANKL treatment significantly reduced cortical bone erosions, and these treatment-associated structural changes were readily detected by BRAVO.

**Conclusion:**

By integrating longitudinal gene expression profiling with high-resolution structural analysis, this study defines coordinated phases of inflammation-driven bone damage and reactive bone formation in experimental inflammatory arthritis. BRAVO provides a robust and sensitive approach for quantifying erosion-associated cortical surface changes and evaluating therapeutic effects on structural outcomes.

**Supplementary Information:**

The online version contains supplementary material available at 10.1186/s13075-026-03874-y.

## Introduction

Rheumatoid arthritis (RA) is a chronic inflammatory disease characterized by persistent synovial inflammation that drives progressive structural joint damage [1, 2]. Cartilage erosion and bone erosions can occur early in the disease and represent defining features of RA, contributing substantially to long-term disability. Although erosive lesions may undergo partial repair under effective anti-rheumatic therapy, complete structural restoration is uncommon, and new erosions can develop despite apparent inflammatory control [3–7]. Together, these observations emphasize the importance of early and sensitive detection of erosive change to guide timely, targeted therapeutic interventions aimed at preventing progressive bone damage and promoting structural repair.

Mechanistically, structural progression in RA reflects a sustained imbalance between bone resorption and insufficient repair, predominantly driven by inflammation-induced osteoclastogenesis and osteoclast-mediated bone resorption [8]. The cytokine- and chemokine-rich synovium promote leukocyte recruitment, stromal cell activation and production of matrix-degrading enzymes, while receptor activator of nuclear factor κB ligand (RANKL), a key regulator of osteoclastogenesis, provides a mechanistic link between inflammation and bone resorption [9–12].

Experimental arthritis models have been instrumental in uncovering mechanisms of joint inflammation and structural damage. They provide a controlled setting to dissect how synovial inflammation, bone erosion and subsequent bone remodeling evolve over time and to link these processes to underlying molecular programs. High-resolution micro-computed tomography (µCT) enables three-dimensional visualization of erosive bone lesions and is widely used in experimental arthritis [13–16]. However, erosion severity is often assessed by observer-based scoring or conventional morphometric parameters that may miss subtle erosive changes and can be influenced by physiological or reparative bone growth, complicating interpretation across ages and experimental conditions [17–19]. More robust and sensitive structural readouts are therefore needed to quantify these processes across disease stages and to validate osteoclast-targeting interventions.

To address this need, we developed BRAVO (Bone Roughness Analysis and Visualization Operator), a µCT-based analysis workflow designed to quantify alterations in the cortical bone surface as a readout of erosive lesions. Here, we combined BRAVO with histopathology and kinetic gene expression profiling in the K/BxN serum-transfer-induced arthritis (STIA) model to define the time-dependent relationship between synovial inflammation and structural damage and to characterize distinct erosive and reparative phases during disease progression.

## Materials and methods

### Mice, K/BxN serum-transfer arthritis and treatment

Male C57BL/6J mice were obtained from Janvier Labs. STIA was induced as previously described using serum from arthritic K/BxN mice [20, 21]. Disease progression was monitored three times per week from the day of serum injection by clinical scoring of all paws and by measurement of ankle swelling, as described previously [22]. Clinical arthritis was scored on a scale from 0 to 4 as follows: 0 = normal paws with no visible inflammation, redness or swelling; 1 = mild but definite redness and/or localized swelling limited to the hind or front paw; 2 = moderate swelling and redness extending beyond a single localized area, involving the toes or a larger region of the paw; 3 = severe swelling and redness affecting the entire paw, including the ankle and toes; and 4 = advanced inflammation with severe swelling of the entire paw, often accompanied by joint rigidity, reduced flexibility or visible deformity. Additional details regarding the STIA model are provided in the Supplementary Materials.

For treatment studies, mice received intraperitoneal (i.p.) injections of either 125 µg anti-mouse RANKL antibody (clone IK22/5, BioXcell) or the corresponding rat IgG2a isotype control (clone 2A3, BioXcell), as described in the corresponding figure legends.

Mice were housed under standard conditions (12-hour light/dark cycle, 21 °C, 48% humidity) with food and water provided ad libitum. All animal experiments were conducted at the Laboratory for Experimental Biomedicine, University of Gothenburg, in accordance with approved ethical protocols (5.8.18–09076/2020 and 5.8.18–07352/2025).

### Micro-computed tomography (µCT) acquisition and reconstruction

Hind paw joints were fixed in 4% formalin for 72 h, stored in 70% ethanol, and rehydrated in PBS for 24 h before scanning. Samples were imaged using a Skyscan 1176 µCT system (Bruker) at a voxel size of 9 μm, with scanning parameters of 54 kV, 467 µA, and a 0.2-mm aluminum filter. Each sample was exposed for 880 ms, with X-ray projections acquired at 0.42° intervals over a 180° rotation using four-frame averaging. Three-dimensional image datasets were reconstructed using NRecon software (version 1.6.9.8, Bruker) and DataViewer (version 1.5.0.9, Bruker). Quantitative analyses were performed using CT-Analyzer software (version 1.14.4.1, Bruker), and three-dimensional visualizations used for semi-quantitative scoring were generated using CTVox (version 2.7, Bruker). Additional details regarding µCT acquisition, image processing, and quantitative analysis are provided in the Supplementary Materials.

### Semi-quantitative µCT scoring of bone erosion and formation

Bone erosion and bone formation were assessed using an observer-based semi-quantitative scoring of three-dimensional µCT scans, following an established scoring system [23, 24]. Briefly, bone erosion was scored as follows: 0 = normal, 1 = mild surface roughness, 2 = surface roughness with minor pitting, 3 = pitting, and 4 = full-thickness cortical defects. Bone formation was scored as follows: 0 = no new bone formation, 1 = mild new bone formation, 2 = moderate bone growth, 3 = extensive new bone coverage, and 4 = continuous new bone present across the defect. All semi-quantitative analyses were performed in a blinded manner. For scoring, the hind paw was divided into two regions: (i) the full hind paw, including the calcaneus, centrale, distal tarsals, astragalus, distal tibia and fibula, and metatarsals, and (ii) the isolated calcaneus. Phalanges were excluded because they exhibited minimal arthritic bone erosion. Representative images illustrating the scoring system are shown in Supplementary Fig. 2A, B.

### Quantitative µCT morphometric analysis and erosive morphology index (EMI)

Quantitative µCT morphometric parameters were assessed using CT-Analyzer software, including bone surface area (SA), representing the outer cortical bone surface, and bone volume (BV), representing total bone volume. The erosive morphology index (EMI) was calculated as the ratio of surface area to bone volume (EMI = SA/BV), as previously described [25, 26], providing a normalized measure of cortical bone surface area relative to total bone volume.

### BRAVO-based cortical bone surface roughness analysis

To quantify arthritis-associated changes in the outer cortical bone surface, we applied a surface-based analysis approach using our in-house MATLAB-based Bone Roughness Analysis and Visualization Operator (BRAVO) workflow, which was adapted from previously established methods [16, 27]. Following reconstruction of three-dimensional bone surfaces from µCT datasets, local surface irregularities were assessed by analyzing angular deviations between neighboring surface elements. Distributions of angular deviations were visualized as histograms, and erosion-associated surface irregularities (SI) were quantified as the area under the curve (AUC) above a defined angular threshold (T > 60°).

To enable quantitative comparison across samples and to account for differences in surface size, the AUC (T > 60°) was normalized to the total number of analyzed local surface regions (Surface Area Units [SAU]), yielding a normalized measure of cortical bone surface roughness. All analyses were implemented using an automated, in-house MATLAB workflow optimized for calcaneus bone analysis. Detailed computational procedures are provided in the Supplementary Materials.

### RNA isolation, qPCR and gene expression analysis

Collected hind paws were flash-frozen in liquid nitrogen. Samples were homogenized in TRIzol reagent (Invitrogen, Cat. #15596018) using a FastPrep-24 5G instrument (MP Biomedicals). Total RNA was extracted using the PureLink™ RNA Mini Kit (Invitrogen) following the manufacturer’s protocol for TRIzol-based isolation. Complementary DNA (cDNA) was synthesized using the iScript™ cDNA Synthesis Kit (Bio-Rad).

Quantitative PCR (qPCR) was performed on a QuantStudio™ 6 Pro Real-Time PCR System (Applied Biosystems) using gene-specific primers and SsoAdvanced Universal SYBR Green Supermix (Bio-Rad). Primer sequences are provided in Supplementary Table 1. Each reaction was run in technical duplicates and gene expression levels were normalized using the ΔCt method to the housekeeping gene *Gapdh. Gapdh* was selected because it is widely used as a reference gene in studies of experimental arthritis and inflamed joint tissues [28–31]. Nevertheless, we acknowledge that the use of a single housekeeping gene represents a limitation of the current study.

### Histopathology

The same hind paws that were previously analyzed by µCT were subsequently decalcified and embedded in paraffin for histological processing. Paraffin-embedded hind paws were sectioned sagittally to an appropriate depth in accordance with SMASH guidelines [32] and stained with either haematoxylin and eosin (H&E) or toluidine blue (TB) using standard histological procedures. Histopathological scoring was performed as previously described [22, 26]. Briefly, scoring was performed in the hind paw tarsal region, including the calcaneus, talus, tibiale, centrale, distal tarsal bones and parts of the metatarsals, whereas the tibia and fibula were excluded from the analysis. For each joint, two sagittal sections were evaluated. In each section, synovial inflammation, bone erosion and cartilage erosion were assessed across the evaluated tarsal region. The findings from both sections were then considered together to assign a single representative histological score for each joint.

Synovial inflammation and bone erosion were assessed in H&E-stained sections using a semi-quantitative scale from 0 to 4. Synovial inflammation was scored based on the extent of inflammatory cell infiltration in the periarticular region: 0 = normal; 1 = minimal infiltration; 2 = mild infiltration; 3 = moderate infiltration; and 4 = marked infiltration. Bone erosion was scored on a scale from 0 to 4: 0 = normal, intact bone; 1 = minimal superficial cortical erosions; 2 = localized subchondral erosions with partial cortical penetration; 3 = extensive cortical and trabecular bone erosion with pannus invasion and near-complete cortical breakthrough; and 4 = severe bone destruction with full-thickness cortical defects and marked trabecular bone loss.

Cartilage erosion was evaluated using TB-stained sections and scored from 0 to 4: 0 = no cartilage damage with complete matrix staining; 1 = localized cartilage erosions; 2 = more extensive erosions; 3 = severe erosions; and 4 = complete cartilage loss. All histological analyses were performed in a blinded manner. Slides were digitized using a Zeiss Axioscan 7 slide scanner (Zeiss) and analyzed using Zen software (Zeiss).

### TRAP staining for osteoclasts

Tartrate-resistant acid phosphatase (TRAP) staining was performed on decalcified paraffin-embedded joint sections to identify osteoclasts. Following deparaffinization and rehydration, sections were incubated in 0.2 M acetate buffer for 90 min and then in TRAP buffer containing 0.1 M acetate, 0.3 M sodium tartrate, 20 mg/mL naphthol AS-MX phosphate, 0.1% Triton X-100 and 0.6 mg/mL Fast Red Violet overnight. Sections were counterstained with 0.05% fast green for 30 s to visualize background tissue morphology and slides were digitized using a Zeiss Axioscan 7 slide scanner (Zeiss) and analyzed using Zen software (Zeiss). TRAP-positive cells located on bone surfaces were identified as osteoclasts. Osteoclast abundance was quantified as the number of TRAP-positive cells per millimeter of bone surface. Quantification was performed in the hind paw tarsal region, including the calcaneus, talus, tibiale, centrale, distal tarsal bones and parts of the metatarsals, whereas the tibia and fibula were excluded from the analysis. For each joint, two sagittal sections were evaluated, and the findings from both sections were considered together to assign a representative value for each joint.

### Statistical analysis

Sample sizes were based on prior experience with C57BL/6J mice to ensure adequate power to detect biologically relevant differences. Statistical analyses were performed using GraphPad Prism (Version 11) or R, and all computational analyses were conducted in Python (version 3.12.11).

Group comparisons were performed using the Mann-Whitney U test or Kruskal-Wallis test with appropriate post hoc corrections, as specified in the figure legends. Principal component analysis (PCA) was performed using Orange Data Mining to visualize the relationship between the two input variables included in each analysis. Time-group labels were used to aid interpretation of sample distribution, but the PCA was based on the input variables only. Because each PCA included two variables, the analysis did not introduce additional dimensions beyond the original feature space and was used as a standardized visualization of the combined metrics rather than as a dimensionality reduction approach. Receiver operating characteristic (ROC) analysis was performed using blinded semi-quantitative µCT bone erosion scoring (erosion score ≥ 1) as the reference assessment. The discriminatory performance of BRAVO and EMI was quantified by calculating the area under the ROC curve (AUC). Differences between AUCs were assessed using paired bootstrap resampling to preserve the paired BRAVO and EMI measurements obtained from the same samples. Additional details regarding the statistical analyses are provided in the Supplementary Materials.

Pairwise associations between gene expression and arthritis-related traits (ankle swelling, EMI and BRAVO) were evaluated using Spearman correlation coefficients. Resulting *P* values were adjusted for multiple hypothesis testing using the Benjamini-Hochberg false discovery rate (FDR) procedure. In heatmap visualizations, correlations that did not remain significant after FDR correction (q ≥ 0.05) are denoted by “¬*”. For time-course analyses, ΔΔCt values were calculated relative to non-arthritic controls and averaged across mice at each time point to generate gene-level mean expression trajectories.

Data are presented as mean ± SEM, as indicated. A two-sided *P* value < 0.05 was considered statistically significant unless otherwise specified. For analyses involving multiple testing, *P* values were adjusted using the Benjamini-Hochberg false discovery rate (FDR) procedure, with *q* ≥ 0.05 considered non-significant.

## Results

### Kinetic histopathological assessment of joints during progression of K/BxN arthritis

**Fig. 1:**
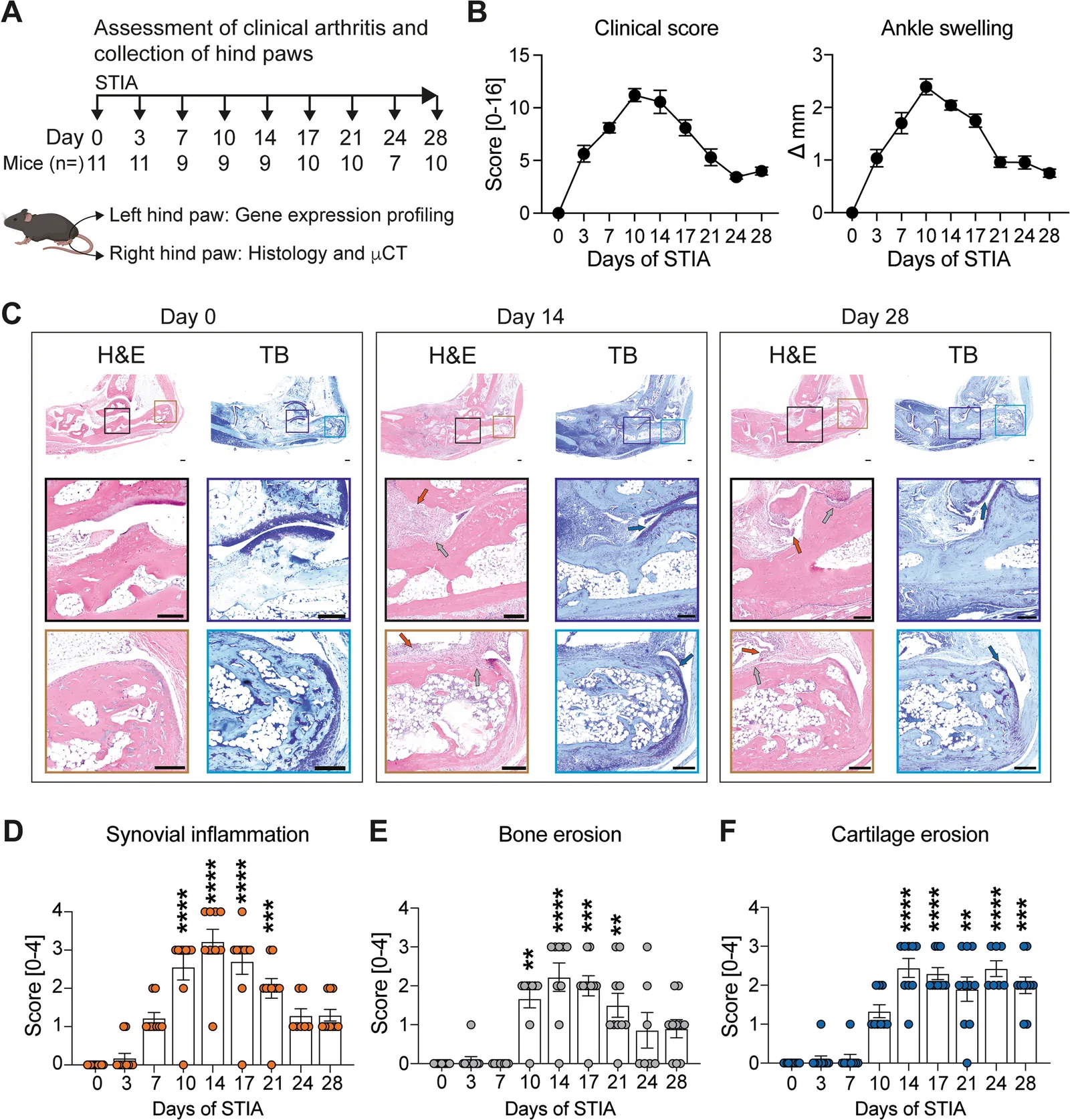
Kinetic assessment of tissue pathology during the course of STIA. **A** Schematic overview of the experimental setup indicating time-points for collection of hind paws used in downstream analysis. N equals numbers of mice terminated at each time-point. **B** Clinical score and ankle swelling in male C57BL/6J mice at different time-points during the course of STIA (*n* ≥ 7 per group, see **A**). See Supplementary Figure 1 for individual mouse data points. **C** Representative haematoxylin and eosin (H&E) and toluidine blue (TB) images of hind paws used for histological analysis of synovial inflammation, bone erosion and cartilage erosion. Orange arrows indicate areas of synovial inflammation, gray arrows indicate areas of bone erosion, and blue arrows indicate areas of cartilage erosion. Scale bars, 200 μm. The representative images correspond to the following histological scores: Day 0 (synovial inflammation = 0, bone erosion = 0, cartilage erosion = 0), Day 14 (synovial inflammation = 3, bone erosion = 3, cartilage erosion = 2), and Day 28 (synovial inflammation = 1, bone erosion = 1, cartilage erosion = 3). **D**-**F** Quantification of synovial inflammation, bone erosion, and cartilage erosion in mice shown in (**A**). Each symbol in **D**, **E**, and **F** represents an individual mouse. Bars represent mean ± SEM. Statistical significance in **D**-**F** was determined by Kruskal-Wallis test followed by Dunn’s multiple comparisons test, with each time point compared to Day 0. Only statistically significant comparisons are indicated in the figures. **P < 0.01, ***P < 0.001, ****P < 0.0001

The K/BxN STIA model is a well-established and widely used experimental model of inflammatory arthritis, with distinct features of inflammation-induced cartilage erosion, bone erosions and reactive bone remodeling [9, 18, 33, 34]. To characterize the time-dependent relationship between synovial inflammation and structural joint damage, we subjected C57BL/6J mice to STIA (Fig. 1A, B and Supplementary Fig. 1) and performed histopathological assessment of hind paws at different time points during disease progression. Synovial inflammation was first observed on day 7, preceding the first detectable signs of bone and cartilage erosion on day 10 (Fig. 1C-F). Synovial inflammation and bone erosion peaked on day 14 and declined progressively until day 28, consistent with inflammatory resolution and bone remodeling. In contrast, cartilage erosion persisted throughout the disease course, suggesting a lack of cartilage repair (Fig. 1F).

Collectively, these data indicate that synovial inflammation precedes structural joint damage and that cartilage loss can persist despite clinical improvement, mirroring key features of inflammatory arthritis in patients [35].

### Three-dimensional µCT assessment of bone remodeling during arthritis progression

**Fig. 2:**
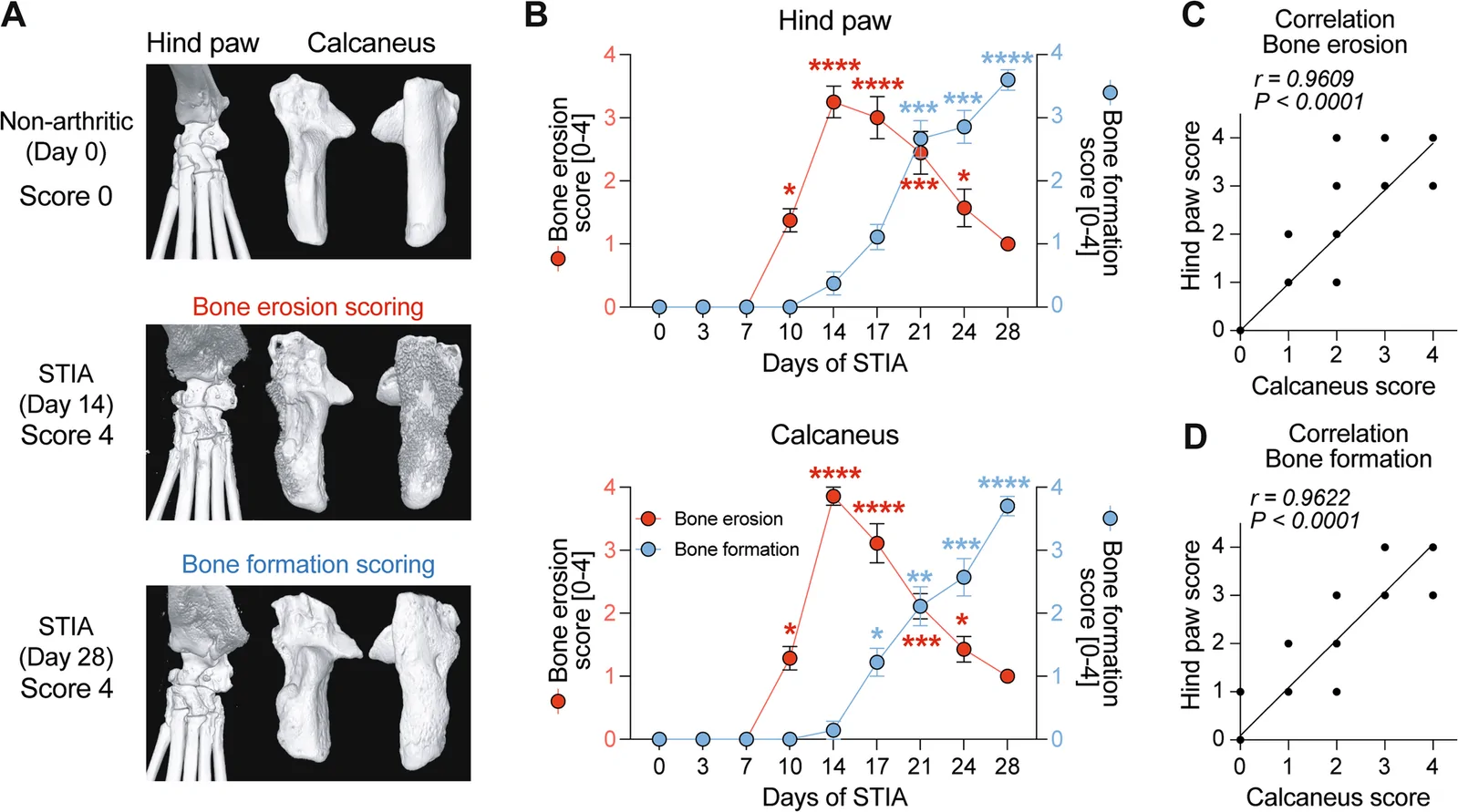
Semi-quantitative scoring of three-dimensional µCT scans reveals distinct phases of bone erosion and formation during STIA. **A** Representative images illustrating of scoring scale used to assess bone erosion and bone formation in the hind paw and the corresponding calcaneus in the STIA model. The complete scoring scale is shown in Supplementary Figure 2A, B. **B** Scoring of bone erosion and bone formation in the whole hind paw and the isolated calcaneus at different time points during STIA (*n* ≥ 7 per group; see Fig. 1A). **C**, **D** Spearman’s correlation analysis of bone erosion (**C**) and bone formation (**D**) scores between the hind paw and corresponding isolated calcaneus, calculated across the full experimental time course using matched hind paw and calcaneus samples. Correlation coefficients (r) and two-tailed *P* values are shown (*P* < 0.0001). Graphs represent mean ± SEM. Statistical significance in **B** was determined by Kruskal-Wallis test followed by Dunn’s multiple comparisons test, with each time point compared to Day 0. Only statistically significant comparisons are indicated in the figures. *P < 0.05, **P < 0.01, ***P < 0.001, ****P < 0.0001

To enable three-dimensional assessment of joint-associated bone remodeling, including features that are less readily captured by two-dimensional histology (such as reparative bone formation), we used µCT imaging during STIA progression and resolution and assessed bone erosion and formation in the hind paw and the isolated calcaneus by semi-quantitative scoring (Fig. 2A, Supplementary Fig. 2A, B).

Bone erosion scores peaked on day 14 of STIA in both the hind paw and the calcaneus (Fig. 2B, Supplementary Fig. 2C), with initial signs of bone erosion detected on day 10, consistent with our histopathological findings (Fig. 1E). By comparison, bone formation was first detected on day 17 post serum injection, approximately three days after peak erosion, and increased progressively to reach maximal levels on day 28 (Fig. 2B, Supplementary Fig. 2D). This sequential pattern is consistent with reparative remodeling occurring as a response to inflammatory bone damage [36, 37].

Notably, erosion and bone formation followed similar time-dependent patterns in both the hind paw and the calcaneus. Accordingly, erosion and formation scores in these regions were strongly correlated (Fig. 2C, D), indicating that the calcaneus reflects overall joint-associated bone remodeling during arthritis. These findings support the use of the calcaneus as a representative bone for µCT-based assessment of bone remodeling in the STIA model.

### Implementation of BRAVO for quantitative analysis of arthritis-induced bone erosions

**Fig. 3:**
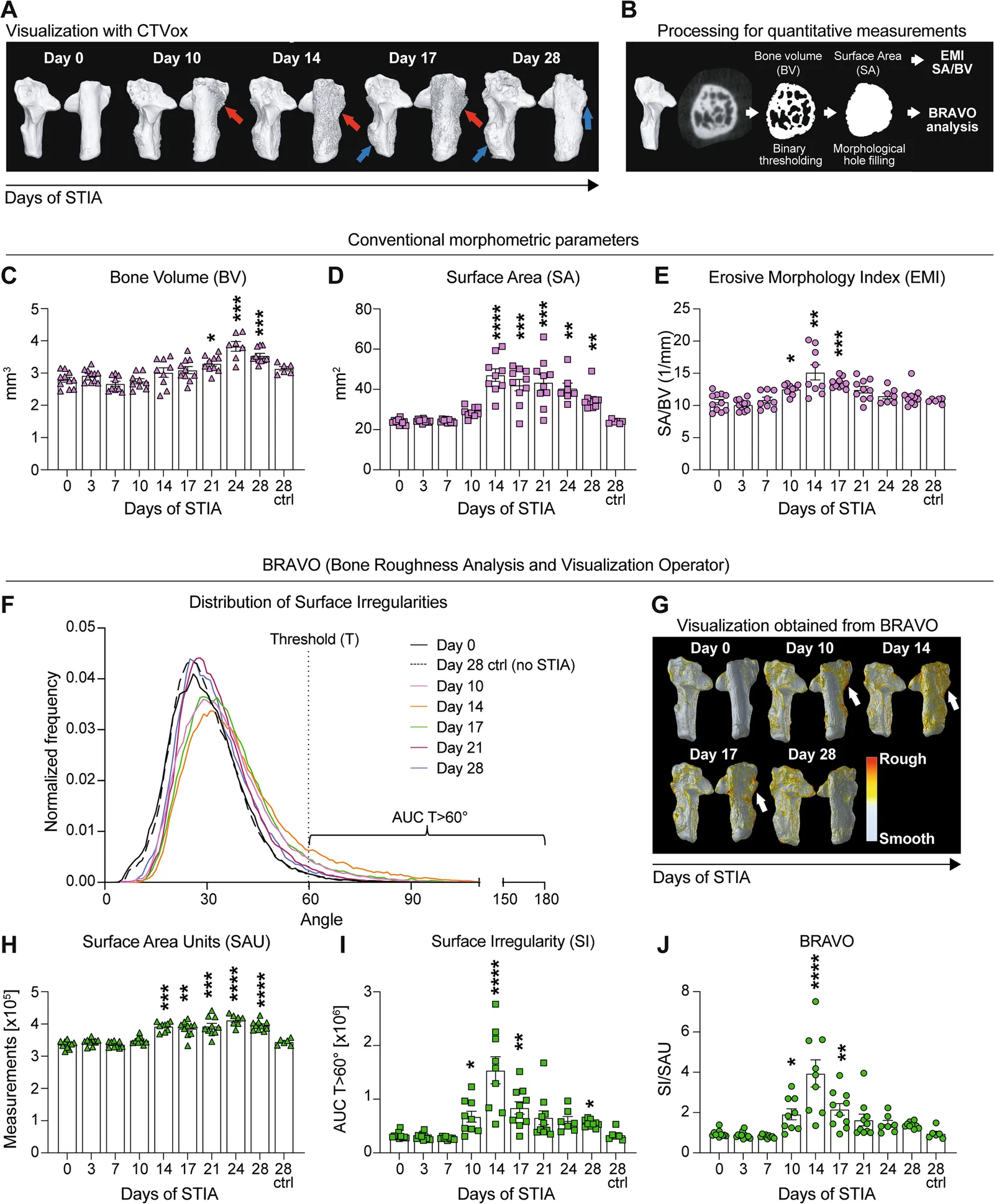
Quantitative µCT-based assessment of cortical bone remodeling during STIA using conventional morphometrics and the BRAVO-based workflow. **A** Representative µCT images of the isolated calcaneus across selected time points during STIA, illustrating changes in bone surface. Red arrows indicate areas of bone erosions and blue arrows indicate areas of bone formation. Images generated using the CTVox software from Bruker. **B** Representative workflow illustrating image processing used for quantitative analysis. Bone volume (BV) was calculated from binary images following thresholding, whereas surface area (SA) and BRAVO analyses were performed after morphological hole filling. See Supplementary Materials for detailed image-processing procedures. **C**-**E** Calculated measurements of bone volume, surface area and Erosive Morphology Index (EMI) across experimental time points during STIA (*n* ≥ 7 per group; see Fig. 1A; Day 28 no STIA controls *n* = 6 [Day 28 ctrl]). **F** Distribution of local roughness across bone surfaces. Control samples (black) peak before 30°, whereas arthritic bones (colored) shift rightward with more values above the ~60° threshold, indicating increased surface irregularity. **G** Heat maps generated from our BRAVO-workflow in MATLAB visualizing areas of increased surface roughness (red) during STIA. White arrows indicate areas of increased roughness (erosions). **H**, **I** The BRAVO-derived measurements Surface Area Units (SAU) (**H**) and Surface Irregularity (SI) (**I**) during STIA. SI is calculated as the area under the curve of angles > 60° (*n* ≥ 7 per group, see Fig. 1A, Day 28 ctrl *n*=6). **J** BRAVO quantification of calcaneus based on normalizing the SI to SAU (SI/SAU) across experimental time points during STIA (*n* ≥ 7 per group, see Fig. 1A, Day 28 ctrl *n*=6). Each symbol in **C**, **D**, **E**, **H**, **I** and **J** represents an individual mouse. Bars represent mean ± SEM. Statistical significance in **C**-**E** and **H**-**J** was determined by Kruskal-Wallis test followed by Dunn’s multiple comparisons test, with each time point compared to Day 0. Only statistically significant comparisons are indicated in the figures. *P < 0.05, **P < 0.01, ***P < 0.001, ****P < 0.0001

Although semi-quantitative scoring provides an overview of arthritis-associated bone changes, more precise quantitative analyses are needed. Therefore, we performed µCT-based morphometric analyses on the calcaneus to quantify joint-associated bone remodeling during STIA. Conventional morphometric parameters commonly used to quantify arthritis-related changes at the cortical surface [25, 26, 38, 39] were first analyzed, including bone surface area (SA), reflecting the outer cortical surface, bone volume (BV), representing total bone volume, and the derived erosive morphology index (EMI), which normalizes surface area to bone volume (SA/BV) (Fig. 3A, B).

Surface area increased during early disease and peaked on day 14 of STIA, consistent with active erosive remodeling, whereas bone volume increased at later stages, coinciding with disease resolution and bone formation (Fig. 3C, D, Supplementary Fig. 3A). EMI also peaked on day 14 (Fig. 3E), showing a 1.5-fold increase compared with non-arthritic animals (day 0) (Supplementary Fig. 3B), before declining towards non-arthritic control levels by day 28. This pattern closely mirrored semi-quantitative erosion scores (Fig. 2B), confirming that normalization of surface area to bone volume can capture erosion-associated structural changes during STIA progression.

While EMI captured the overall pattern of bone erosion during STIA, the magnitude of change was modest. Therefore, we applied our in-house BRAVO analysis workflow, which quantifies irregularities in otherwise smooth bone surfaces to improve detection of arthritis-associated erosive lesions.

Histogram and heat-map visualizations generated by BRAVO revealed pronounced surface irregularities, corresponding to rough cortical bone areas, in arthritic ankles during STIA compared with non-arthritic controls (Fig. 3F, G, Supplementary Fig. 3A). Quantitative BRAVO measurements were derived by normalizing measures of surface irregularity to the number of analyzed local surface regions (Fig. 3H, I). BRAVO values markedly increased on days 10 and 14 (Fig. 3J), corresponding to 2.0-fold and 4.4-fold increases, respectively, compared with non-arthritic animals (day 0) (Supplementary Fig. 3C). Importantly, BRAVO demonstrated clearer discrimination between disease stages than EMI. This observation was further supported by ROC analysis using blinded semi-quantitative µCT bone erosion scoring as the reference assessment (AUC = 0.973 vs. 0.866; *P* = 0.0006; Supplementary Fig. 3D).

### BRAVO provides robust and age-independent quantification of bone remodeling

**Fig. 4:**
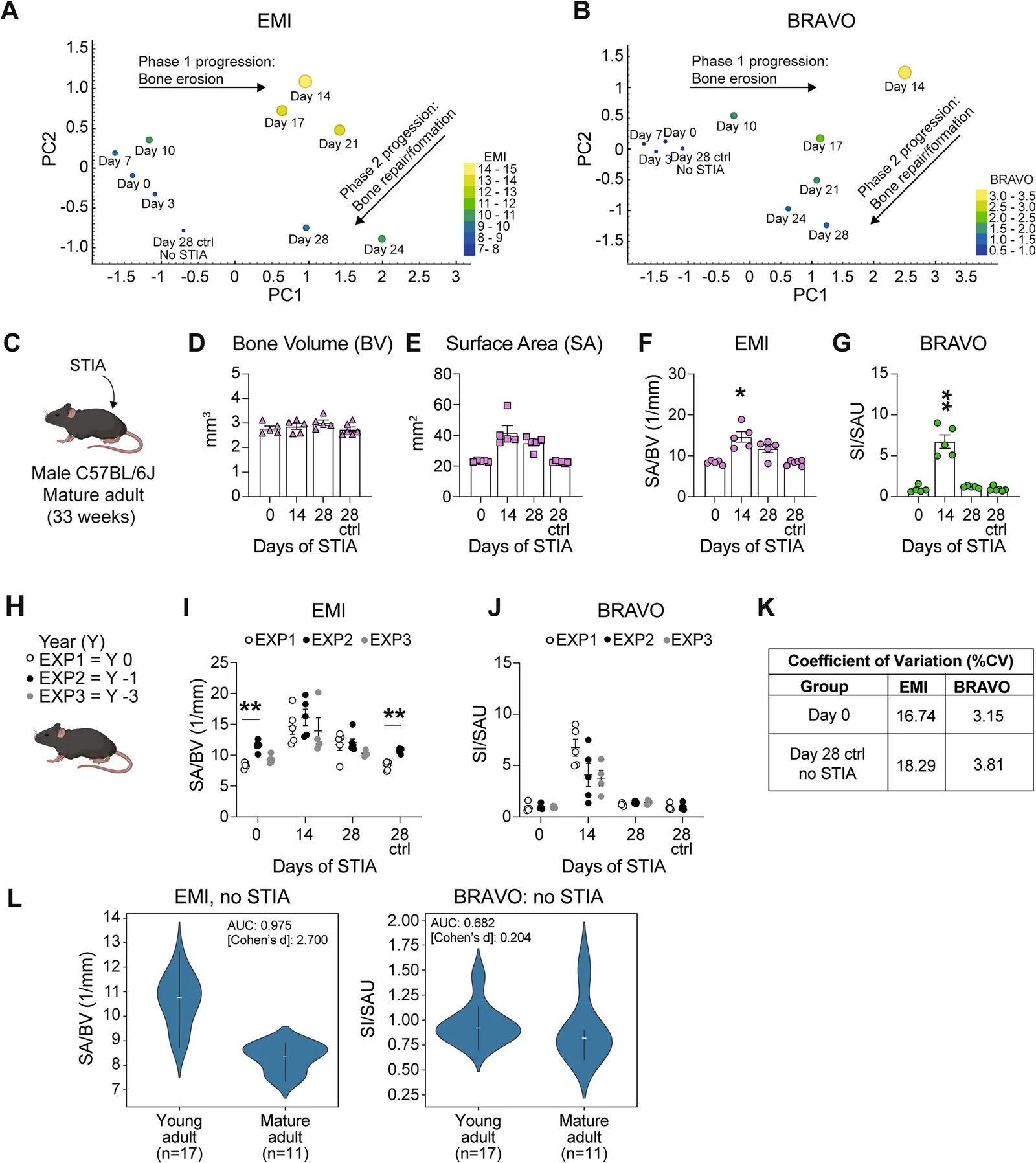
BRAVO provides robust and age-independent quantification of cortical bone remodeling during inflammatory arthritis. **A**, **B** Principal Component Analysis (PCA) of EMI and BRAVO analyses. EMI PCA was generated using bone volume and surface area as input variables, whereas BRAVO PCA was generated using surface irregularities and surface area units. **C** Schematic overview of a 33-week-old male C57BL/6J mouse. **D**-**F** Quantitative measurements of bone volume (BV), and surface area (SA) and EMI across different time points during STIA (*n* ≥ 5 per group; see Supplementary Figure 4A). **G** BRAVO quantification across different time points during STIA (*n* ≥ 5 per group, see Supplementary Figure 4A). **H** Schematic overview of the three independent STIA experiments: EXP1 (performed in 2025; mice aged 33 weeks), EXP2 (2024; 12 weeks), and EXP3 (2022; 12 weeks). **I**-**J** Average value of EMI and BRAVO from the three independent STIA experiments (EXP1-3) across 3 years (*n* ≥ 5 per EXP; see Fig. 1A and Supplementary Figure 4A). **K **%CV for EMI and BRAVO of non-arthritic (no STIA) mice on day 0 and day 28. **L** Violin plots with embedded box plots showing BRAVO and EMI distributions in healthy non-arthritic (no STIA) young adult (12-16 weeks of age) and mature adult (33-37 weeks of age) mice (*n* ≥ 11 per group). For each comparison, group separability was quantified using the area under the ROC curve (AUC), defined as the probability that randomly selected sample is correctly ranked by the metric. Cohen’s d was calculated as the standardized mean difference, defined as the difference between group means divided by the pooled standard deviation, describing how distinct the two groups are. Each symbol in **D**, **E**, **F** and **G** represents an individual mouse. Graphs represent mean ± SEM. Statistical significance in **D**-**G**, **I** and **J** was determined by Kruskal-Wallis test followed by Dunn’s multiple comparisons test, with each time point compared to Day 0. Only statistically significant comparisons are indicated in the figures. *P < 0.05, **P < 0.01

To further compare how EMI and BRAVO capture changes in bone remodeling over the course of arthritis, we performed principal component analysis (PCA) separately using key metrics derived from each analysis workflow. PCA of EMI and BRAVO revealed two distinct structural phases during STIA. Early time points (days 3–7) clustered with non-arthritic controls (day 0 and non-arthritic day 28), whereas samples from peak arthritis (day 14) were distinctly separated along PC1, consistent with maximal bone erosion (Fig. 4A, B). As disease progressed, later time points (days 17–28) shifted away from the erosion-associated cluster towards a second trajectory, consistent with a transition from bone erosion to bone formation and remodeling. Notably, BRAVO provided improved resolution of disease stages within the remodeling phase, with clear separation of days 10, 14, 17 and 21, whereas these time points largely overlapped when assessed using EMI (Fig. 4A, B).

BRAVO showed minimal separation between non-arthritic controls on days 0 and 28, indicating that it was less influenced by age-related differences in bone morphology compared with EMI (Fig. 4A, B). To assess the influence of age on these analytical approaches, we subjected mature adult mice (33 weeks old), an age at which physiological bone growth is complete [40], to STIA and evaluated bone remodeling on days 14 and 28 post-serum injection (Fig. 4C, Supplementary Fig. 4A, B).

For EMI-derived parameters, bone volume remained largely unchanged between healthy controls and arthritic mice, consistent with limited net bone turnover in mature adult animals [41], whereas surface area, and consequently EMI, was increased on both days 14 and 28 (Fig. 4D-F). The persistent elevation in surface area on day 28 coincided with increased bone formation detected by semi-quantitative scoring (Supplementary Fig. 4C, E), suggesting that the non-significant trend towards elevated EMI at this time point may reflect reactive bone formation rather than persistent bone erosion. By comparison, BRAVO-based analysis revealed a marked increase in cortical bone erosions on day 14 that returned to levels comparable to non-arthritic mice by day 28 post-serum injection (Fig. 4G and Supplementary Fig. 4C, D), consistent with erosion scores obtained by semi-quantitative scoring (Supplementary Fig. 4E).

Given the potential age- and bone formation-related influence on EMI, we next evaluated the robustness of EMI and BRAVO across three independent STIA experiments performed 1 to 2 years apart using cohorts that differed in age. These experiments were not intended to serve as biological replicates but rather to assess the robustness of each analytical approach across independent experimental cohorts. BRAVO-based analysis demonstrated substantially lower variability than EMI in non-arthritic animals, indicating greater robustness to cohort-dependent differences in bone morphology (Fig. 4H-K). In arthritic animals, some variation in BRAVO values was observed between experimental cohorts, most likely reflecting differences in disease severity rather than baseline bone characteristics.

Age-stratified analysis revealed a strong age dependency for EMI, with near-complete separation between young adult and mature adult mice (Fig. 4L). By contrast, BRAVO exhibited minimal age-related variation, supporting its use as an age-independent structural metric (Fig. 4L). Further evidence for EMI’s age dependency was provided by moderate separation within young adult mice but minimal separation among mature adult mice (Supplementary Fig. 4F, G).

Together, these results demonstrate that BRAVO is less influenced by age-related differences in bone morphology than EMI and exhibits greater stability across independent experimental cohorts. In contrast to EMI, which varies substantially with age-dependent changes in bone volume, BRAVO showed comparable baseline values across healthy animals and can therefore more reliably capture disease-associated changes in cortical surface irregularity during disease progression, establishing it as a robust metric for quantifying bone structural changes in inflammatory arthritis.

### Kinetic gene expression profiling characterizes molecular changes during arthritis progression

**Fig. 5:**
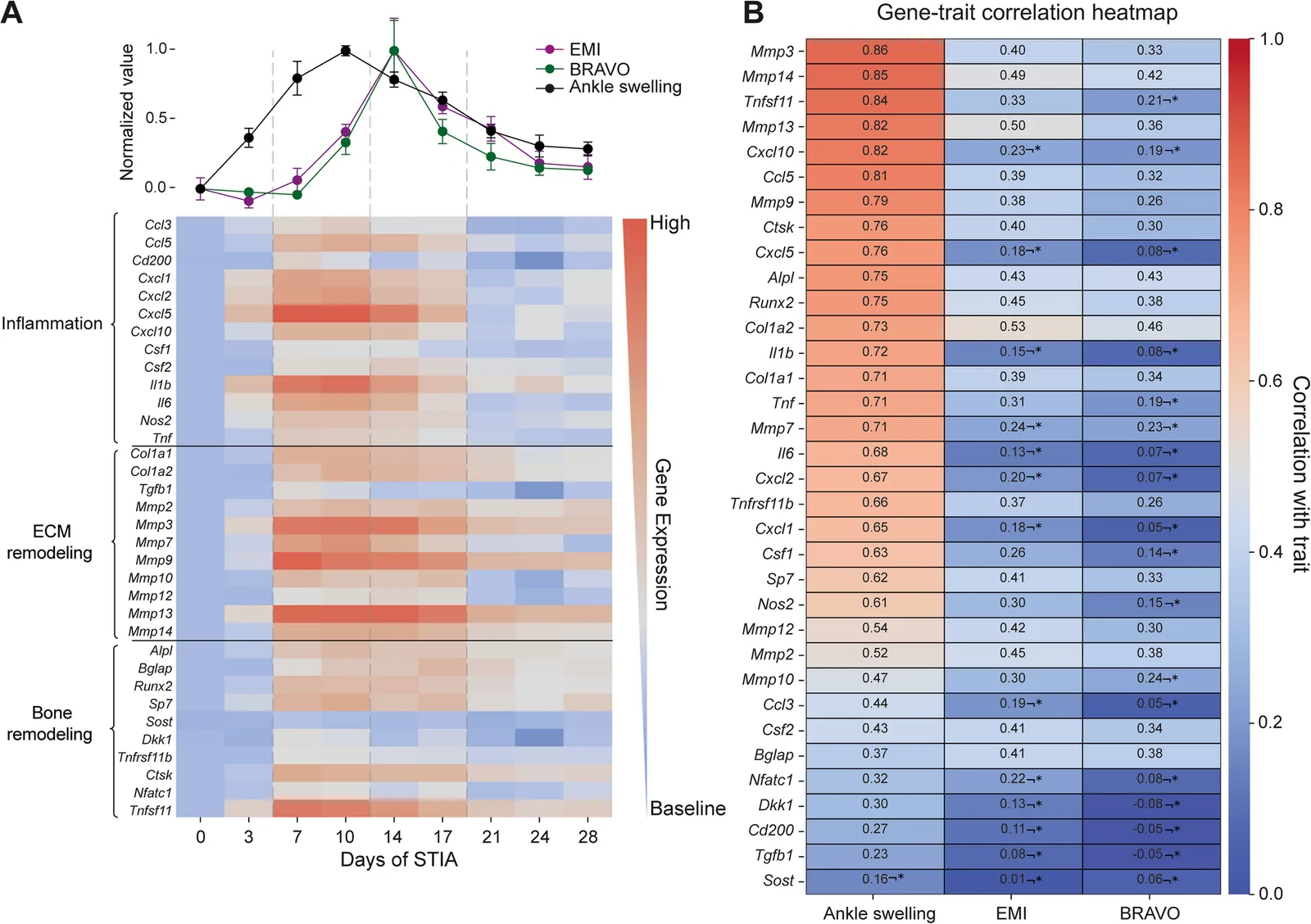
Kinetic joint transcriptional profiling links inflammatory and bone-remodeling programs to clinical severity and structural bone changes during STIA. **A** Time-course heatmap showing mean normalized gene expression (-ΔΔCt) at each experimental time point (*n* ≥ 7 per group; see Fig. 1A), with genes grouped according to predefined functional categories. Arthritis traits (ankle swelling, EMI, and BRAVO) are shown for reference. Gene expression values were normalized using the ΔΔCt method relative to non-arthritic (day 0) controls with Gapdh as the reference gene. For visualization, sign-flipped values (−ΔΔCt) were plotted so that increased expression corresponds to higher heatmap intensity. **B** Gene-trait correlation heatmap showing Spearman correlation coefficients between gene expression levels and individual arthritis traits, calculated across the full experimental arthritis timeline. *P*-values were adjusted using the Benjamini-Hochberg false discovery rate (FDR) procedure. Correlations that did not remain significant after FDR correction (q ≥ 0.05) are marked with “¬*”, whereas unlabeled cells indicate statistically significant associations

To complement our structural analysis, we further characterized the molecular programs underlying inflammation-driven bone erosion and subsequent remodeling by performing kinetic gene expression analysis of arthritic hind paws during STIA (Fig. 5A). Expression of inflammatory mediators (e.g., *Tnf*,* Il1b*,* Il6*) and leukocyte-recruiting chemokines (e.g., *Ccl5*,* Cxcl1*,* Cxcl10*) closely mirrored clinical disease severity, peaking between days 7 and 10 post-serum injection, followed by gradual decline during disease resolution (Fig. 5A, Supplementary Fig. 5A, Supplementary Table 2 A). In parallel, genes associated with osteoclast activity and osteoclastogenic signaling (e.g., *Ctsk* encoding Cathepsin K and *Tnfsf11* encoding RANKL) were upregulated on days 7 and 10 (Fig. 5A, Supplementary Fig. 5B, Supplementary Table 2B), coinciding with peak inflammation and the initiation of bone destruction, before progressively declining during resolution.

Matrix metalloproteinases (MMPs), key mediators of extracellular matrix (ECM) degradation and joint remodeling [42], also exhibited distinct stage-specific expression patterns (Fig. 5A, Supplementary Fig. 5C, Supplementary Table 2 C). *Mmp2*,* Mmp7* and *Mmp9* were upregulated early in disease, consistent with roles in initiating extracellular matrix degradation, cartilage erosion, as well as leukocyte and osteoclast recruitment [42, 43], whereas *Mmp3*,* Mmp10*,* Mmp12*,* Mmp13* and *Mmp14* peaked at later stages, aligning with bone remodeling processes and coinciding with measurable changes in cartilage integrity and bone structure (Fig. 1E, F). In contrast, osteoblast-associated markers (*Alpl* encoding alkaline phosphatase and *Bglap* encoding osteocalcin) displayed a delayed expression profile (Fig. 5A, Supplementary Fig. 5B, Supplementary Table 2C), remaining elevated beyond peak clinical arthritis and reaching maximal levels around day 17, consistent with the observed shift towards bone formation (Fig. 2B). Upregulation of type I collagen genes (*Col1a1*,* Col1a2*) during mid-stage disease coincided with the onset of reactive bone formation and is consistent with activation of matrix deposition in response to inflammatory bone damage. This was accompanied by increased *Tgfb1* expression (Supplementary Fig. 5C), further supporting activation of tissue-remodeling programs during disease progression.

Together, these findings provide molecular context for the structural changes observed during disease progression and highlight inflammatory, bone-remodeling, and extracellular matrix remodeling programs associated with distinct stages of STIA.

### Transcriptional programs correlate with clinical severity and structural bone remodeling

To determine how the observed transcriptional changes relate to clinical disease and structural bone changes across the arthritis course, Spearman correlation analyses were performed between gene expression levels and arthritis-associated traits across the full experimental timeline and visualized in a gene-trait correlation heatmap (Fig. 5B).

Overall, the genes showing the strongest correlations were predominantly associated with immune cell recruitment, extracellular matrix degradation and bone remodeling, highlighting these processes as prominent features of joint pathology during STIA. Correlations were generally stronger with ankle swelling than with quantitative structural bone metrics, consistent with ankle swelling reflecting cumulative inflammatory activity across the disease course. Notably, EMI and BRAVO showed comparatively stronger associations with bone and matrix remodeling genes than with chemokines, consistent with these metrics capturing tissue remodeling-related outcomes.

Among correlated genes, the chemokines *Cxcl10* and *Ccl5* emerged as strong correlates of ankle swelling, in line with their reported involvement in inflammatory cell recruitment in experimental arthritis [44, 45]. Several MMPs also showed robust associations with ankle swelling, consistent with their involvement in extracellular matrix degradation, tissue remodeling and joint destruction during arthritis progression [23, 43].

Within bone remodeling-associated genes, *Tnfsf11* (RANKL) was among the strongest correlates of ankle swelling, consistent with its reported upregulation during STIA [23]. By contrast, *Tnfsf11* showed weak or non-significant correlations with quantitative measures of bone erosion, consistent with transcriptional activation of osteoclastogenic pathways preceding measurable structural bone changes. In line with this, structural associations were generally stronger for genes involved in tissue remodeling and bone turnover, including MMPs (e.g., *Mmp3*,* Mmp9 and Mmp14*), osteoclast markers (e.g., *Ctsk*) and osteogenic/matrix genes (e.g., *Col1a2*,* Alpl* and *Runx2*). Together, these findings indicate that early inflammatory and osteoclastogenic transcriptional programs precede measurable structural bone changes during STIA.

### BRAVO captures treatment-induced suppression of bone erosion during STIA

**Fig. 6:**
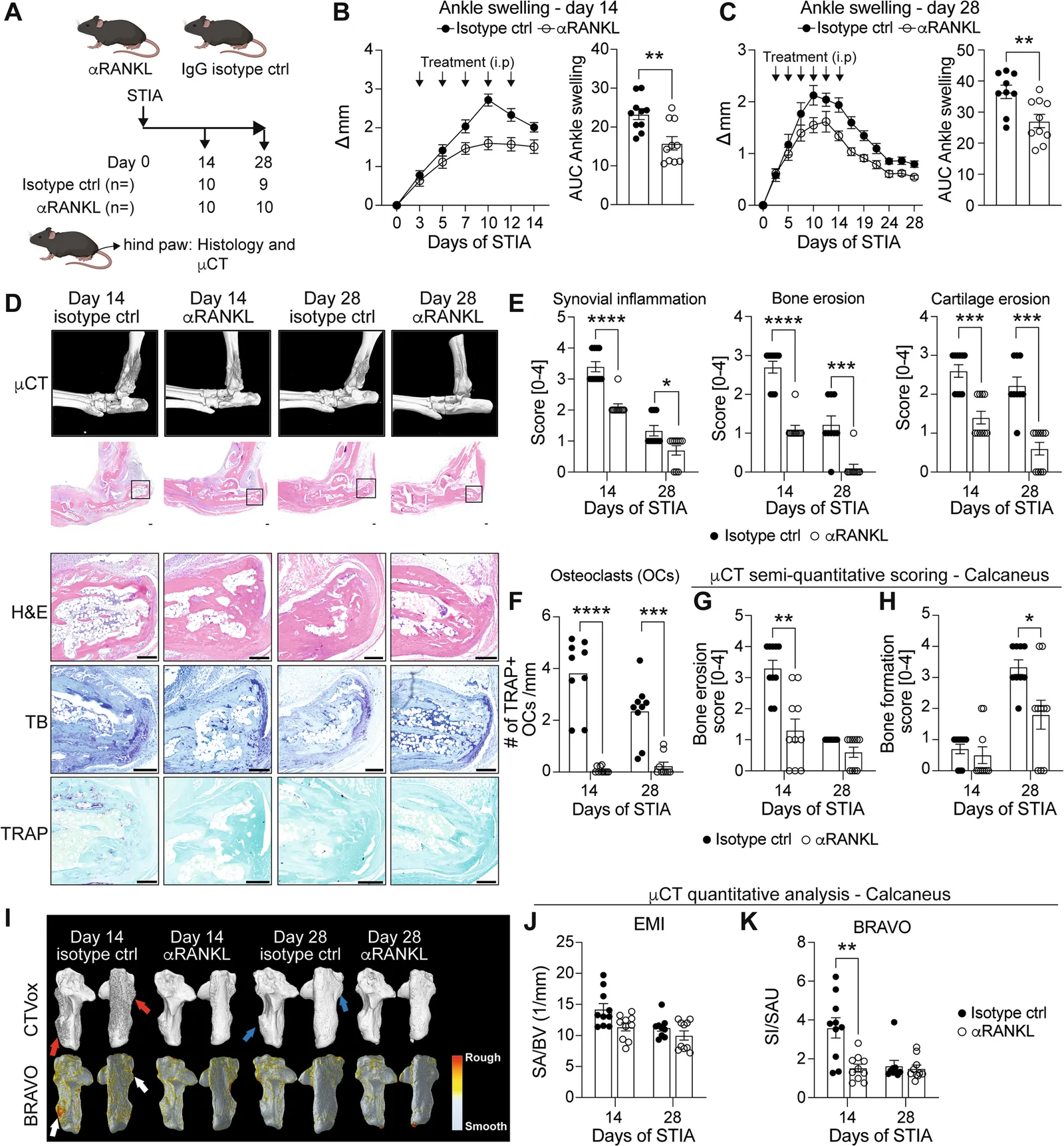
BRAVO detects treatment-induced suppression of cortical bone erosion during STIA. **A** Schematic overview of experimental setup of treated mice (anti-RANKL [αRANKL] vs. IgG isotype ctrl) indicating time points and collection of hind paws for further downstream analysis. N equals numbers of mice terminated per time point. **B**, **C** Ankle swelling in αRANKL-treated and IgG isotype-treated mice during STIA progression of mice terminated either on day 14 or day 28, separately. Treatment was initiated on day 3 after STIA induction. Mice terminated on day 14 received treatment on days 3, 5, 7, 10 and 12, whereas mice terminated on day 28 received treatment on days 3,5,7,10, 12 and 14. Arrows indicate intraperitoneal (i.p) antibody injection (*n* ≥ 9 per group, see **A**). Differences in arthritis severity were quantified using the area under the curve (AUC). **D**-**F** Representative µCT, H&E, TB and TRAP images (**D**) used for histological analysis of synovial inflammation, bone erosion, cartilage erosion (**E**) and TRAP-positive osteoclasts (**F**) in arthritic joints of αRANKL and IgG isotype-treated animals on day 14 and day 28 after STIA (*n* ≥ 9 per group, see **A**). Scale bars, 200 μm. **G**, **H** Semi-quantitative scoring of bone erosion (**G**) and bone formation (**H**) of the isolated calcaneus between treatment groups at day 14 and day 28 after STIA (*n* ≥ 9 per group, see **A**). **I** Representative µCT images of the calcaneus from αRANKL and IgG isotype-treated mice generated using CTVox and the BRAVO workflow. Red arrows indicate areas with bone erosion. Blue arrows indicating areas with bone formation. White arrows indicating areas of increased cortical surface roughness. **J**, **K** Quantitative µCT analysis of the calcaneus from αRANKL or IgG isotype-treated mice at day 14 and day 28 after STIA using EMI (**J**) and BRAVO (**K**) (*n* ≥ 9 per group, see **A**). Graphs represent mean ± SEM. Statistical significance in **B**, **C**, **E**, **F**, **G**, **H**, **J** and **K** was determined by Mann-Whitney U test. Only statistically significant comparisons are indicated in the figures. *P < 0.05, **P < 0.01, ***P < 0.001, ****P < 0.0001

Given the strong association of *Tnfsf11* with disease severity, we next evaluated whether RANKL inhibition alters inflammation and bone remodeling during STIA (Fig. 6A). Based on the kinetics of *Tnfsf11* expression (Supplementary Fig. 5B), treatment of mice with either a RANKL-targeting antibody or the corresponding isotype control was started three days after STIA induction (Fig. 6A).

Clinical assessment showed that anti-RANKL treatment significantly reduced arthritis severity compared with controls (Fig. 6B, C, Supplementary Fig. 6A, B), which was supported by reduced histopathological signs of synovial inflammation, bone erosion and cartilage erosion (Fig. 6D, E). In addition, quantification of TRAP-positive osteoclasts at the cortical bone surface demonstrated reduced osteoclast numbers following anti-RANKL treatment (Fig. 6D, F). Semi-quantitative scoring of µCT scans further demonstrated significant reductions in bone erosion on day 14 and subsequent bone formation on day 28 in both the hind paw and the isolated calcaneus following anti-RANKL treatment (Fig. 6G, H, Supplementary Fig. 6C, D).

Quantitative µCT analysis revealed reduced bone erosion on day 14 in anti-RANKL-treated animals using BRAVO (Fig. 6K, Supplementary Fig. 6F), which was supported by BRAVO-generated heat maps illustrating reduced erosion-associated cortical surface irregularity (rough areas) in anti-RANKL–treated mice (Fig. 6I). Although the surface area parameter differed between treatment groups (Supplementary Fig. 6E), no significant treatment effect was detected using the integrated EMI analysis (Fig. 6J).

Together, these findings show that RANKL blockade attenuates synovial inflammation and joint destruction and establish BRAVO as a robust quantitative readout for detecting treatment-induced changes in cortical bone erosion during inflammatory arthritis.

## Discussion

Joint destruction is a major cause of long-term disability in RA, and preventing erosive progression remains a key therapeutic goal [4, 46]. In this study, we developed and applied BRAVO, a µCT analysis workflow that quantifies cortical bone surface alterations as a sensitive measurement of erosive change during arthritis. Using the K/BxN STIA model, we resolved the time-dependent relationship between synovial inflammation and structural damage, demonstrating that inflammation precedes measurable cartilage and bone destruction and that cartilage loss persists despite clinical improvement. Moreover, anti-RANKL treatment attenuated both disease severity and cortical bone erosions, and BRAVO readily detected these treatment-associated structural changes, underscoring the enhanced sensitivity of BRAVO for evaluating osteoclast-targeting interventions.

A major outcome of this work is the performance of BRAVO as a robust structural readout of erosive damage. Compared with EMI, which was influenced by age and showed greater inter-experimental variability, BRAVO more reliably detected arthritis-induced cortical bone surface alterations and better separated disease stages and treatment groups. Beyond experimental studies, clinical assessment of erosions by radiography and advanced imaging such as MRI or CT-based techniques is often reported using observer-based semi-quantitative scoring, which may limit sensitivity to subtle erosive progression, requires specialized expertise and is both time- and labor-intensive, introducing potential reader-dependent variability and substantial resource demands [6, 47]. A surface-based approach such as BRAVO could, in the future, complement existing imaging readouts by enabling more quantitative assessment of erosive lesions and treatment response.

A prior kinetic study in STIA has profiled joint gene expression during the early inflammatory phase, when clinical scores peak [48]. To our knowledge, this is the first study to combine histopathology, quantitative three-dimensional bone assessment and transcriptional profiling across the full course of STIA. This approach outlines a clear sequence of joint pathology in which inflammatory activity and immune cell-recruiting programs peak early, followed by a matrix-degrading and erosive phase and a later shift towards remodeling-associated signatures. By aligning clinical assessments with tissue-level inflammation, structural readouts and gene expression trajectories, our results help guide selection of time points and endpoints for mechanistic and therapeutic studies, particularly those targeting osteoclast-mediated damage, matrix remodeling or inflammation-driven structural progression.

The osteoclast-inhibiting anti-RANKL antibody denosumab is widely used for treatment of osteoporosis [49] but is not routinely used for RA. Nevertheless, clinical studies have shown that adding denosumab to conventional anti-rheumatic therapy can limit radiographic erosion progression in RA, with less consistent effects on clinical inflammation or cartilage outcomes [50, 51]. In experimental settings, disruption of the RANK-RANKL pathway has likewise been reported to limit structural damage without clearly altering clinical inflammation [9, 11]. By contrast, we found that anti-RANKL treatment reduced structural damage as well as clinical and histological inflammation in STIA, consistent with recent observations in the TNF-transgenic model [52]. This could reflect indirect effects of limiting osteoclastogenesis on inflammatory tissue injury and damage-associated signaling, and/or additional RANKL-dependent mechanisms beyond osteoclast differentiation, which will require further study. Importantly, the timing of our intervention was guided by the kinetic profile of *Tnfsf11* expression together with histological and quantitative µCT readouts, enabling treatment to be initiated during the expected window of high RANKL-associated osteoclastogenic activity and highlighting the value of kinetic analyses for optimizing therapeutic study design.

It should be noted that our transcriptional profiling was based on an expanded but selected candidate gene panel and was not intended to comprehensively define all pathways involved in bone repair or remodeling. Rather, this transcriptional profiling was designed to provide a more balanced kinetic context for inflammation, bone erosion, bone formation and resolution-associated processes during STIA. Future studies incorporating broader transcriptomic and proteomic approaches, together with denser sampling during early disease, will be required to more precisely define the molecular programs linking inflammation to structural change. In addition, the STIA model represents an acute, antibody-mediated arthritis and may not fully recapitulate the chronic synovial pathology of established RA. Although the K/BxN STIA model is a well-established and widely used model of inflammatory arthritis and therefore provides an appropriate setting for benchmarking new structural analysis approaches, our findings are currently limited to this experimental model. Furthermore, all experiments were performed in male mice. Therefore, although BRAVO provided robust quantification of cortical bone erosions in the STIA model, its performance across additional inflammatory arthritis models, non-inflammatory models of joint degeneration, and female animals remains to be evaluated. Future studies in such systems will be important for establishing the general applicability and limitations of the BRAVO workflow. While we observed a distinct reparative remodeling phase following peak erosion, complete structural restoration is uncommon in patients with RA. Therefore, the reactive bone formation in the STIA model likely reflects remodeling connected to erosion, consistent with limited structural repair observed clinically. Nonetheless, BRAVO provides a robust quantitative approach for assessing cortical bone structural changes and detecting treatment-induced alterations in bone structure during experimental inflammatory arthritis.

## Conclusion

In this study, we define the time-dependent relationship between synovial inflammation, bone erosion and subsequent remodeling in experimental inflammatory arthritis. By integrating histology, gene expression and µCT-based structural analysis, we show that BRAVO provides a robust measure of cortical bone erosions and their response to treatment. These findings highlight the value of combining molecular and structural readouts across disease progression and establish BRAVO as a useful tool for assessing erosive pathology in experimental arthritis models.

## Supplementary Information

Supplementary Material 1.

## Data Availability

All data needed to evaluate the conclusions in the paper are present in the paper and/or the Supplementary Materials. Additional data related to this paper may be requested from the authors. The BRAVO MATLAB script is available in GitHub (https://github.com/davemcb94/BRAVO).

## Abbreviations

RA: Rheumatoid Arthritis
RANKL: Receptor activator of nuclear factor κB ligand
µCT: Micro-computed tomography
BRAVO: Bone Roughness Analysis and Visualization Operator
STIA: K/BxN serum-transfer-induced arthritis
i.p.: Intraperitoneal
EMI: Erosive morphology index
ECM: Extracellular matrix
SA: Surface area
BV: Bone volume
SI: Surface irregularities
AUC: Area under the curve
SAU: Surface Area Units
cDNA: Complementary DNA
qPCR: Quantitative PCR
H&E: Haematoxylin and eosin
TB: Toluidine blue
PCA: Principal component analysis
FDR: False discovery rate
SEM: Standard error of mean
IL: Interleukin
TNF: Tumor necrosis factor
CXCL: Chemokine C-X-C motif ligand
CCL: Chemokine C-C motif ligand
MMPs: Matrix metalloproteinases
Tnfsf11: TNF superfamily member 11
Ctsk: Cathepsin K
Alpl: Alkaline phosphatase
Bglap: Bone Gamma-Carboxyglutamate Protein
Col1a1: Collagen Type I Alpha 1 Chain
Col1a2: Collagen Type I Alpha 2 Chain
RUNX2: Runt-related transcription factor 2
TRAP: Tartrate-resistant acid phosphatase
