# Supplementary material for: Inflammation-driven bone erosion and reactive remodeling in experimental arthritis revealed by cortical bone surface analysis
Source: Arthritis Res Ther. 2026 Aug 6;28:155. doi: 10.1186/s13075-026-03874-y (PMC13499316; doi:10.1186/s13075-026-03874-y)
Supplement: Supplementary file 1 [file 13075_2026_3874_MOESM1_ESM.docx]

**Supplementary Materials**

**Inflammation-driven bone erosion and reactive remodeling in experimental arthritis revealed by cortical bone surface analysis**

Anders Nguyen^1^ MSc, David A. McBride^2^ PhD, Miriam Bollmann^1^ PhD, Liam Davidsson^3^ MSc, Nour Dada^1^ MSc, Taymaa Sami^4^ MSc, Lisi Wagener^1^ MSc, Carmen Corciulo^4^ PhD, Muhammad Arif^3,5^ PhD, Linda. C. Johansson^5,6^ PhD, Mattias N.D. Svensson^1^ PhD*

**Affiliations**

^1^Department of Rheumatology and Inflammation Research, Sahlgrenska Academy, Institute of Medicine, University of Gothenburg, Sweden

^2^Calibr-Skaggs Institute for Innovative Medicines, The Scripps Research Institute, La Jolla, CA, USA

^3^Department of Molecular and Clinical Medicine, Sahlgrenska Academy, Institute of Medicine, University of Gothenburg, Sweden

^4^Department of Pharmacology, Sahlgrenska Academy, Institute of Neuroscience and Physiology, University of Gothenburg, Sweden

^5^SciLifeLab, University of Gothenburg, Sweden

^6^Department of Medical Biochemistry and Cell biology, Sahlgrenska Academy, Institute of Medicine, University of Gothenburg, Sweden

*Correspondence to Mattias N. D. Svensson Department of Rheumatology and Inflammation Research, Sahlgrenska Academy, Institute of Medicine, University of Gothenburg, Sweden, 41346 Gothenburg, Sweden, [mattias.svensson@rheuma.gu.se](mailto:mattias.svensson@rheuma.gu.se)

**Supplementary material and methods**

**Mice**

KRN mice on a C57BL/6J background, generously provided by C. Benoist (Harvard University) (1), were bred with non-obese diabetic (NOD) mice obtained from Taconic to generate K/BxN offspring. These mice spontaneously develop arthritis beginning at approximately 4-5 weeks of age. Serum was collected from 10-week-old arthritic K/BxN mice, pooled, and 200 µL was injected intraperitoneally (i.p.) into recipient mice to induce serum-transfer-induced arthritis (STIA), as previously described (2,3).

**μCT image processing and bone quantification**

Following μCT scanning and reconstruction, image processing and quantitative analysis were performed using NRecon, DataViewer, CTAn, and CTVox (Bruker software). Acquired two-dimensional μCT images were first reconstructed into a three-dimensional dataset and spatially aligned to ensure consistent orientation across samples. The calcaneus was then isolated from the hind paw by defining a region of interest (ROI) encompassing only the calcaneus bone.

*Binary thresholding and ROI refinement****.*** For quantitative analysis, grayscale images of the isolated calcaneus were converted into binary images (black and white) by thresholding using the binary selection tool in CTAn together with built-in adaptive median-C algorithm, which calculates a local threshold from the median grayscale value of neighboring pixels and subtracts a constant to account for differences in image intensity. Thresholding was used to segment the foreground (mineralized bone) from the background (non-bone tissue), resulting in binary images where bone volume pixels (voxels) were assigned a value of white and non-bone voxels a value of black.

Following thresholding, several preprocessing steps were applied to refine the binary images:

1. Removal of speckles: Small isolated voxel clusters not connected to the main bone structure were removed to eliminate noise and artifacts from further analysis.
2. ROI shrinking: The region of interest was automatically reduced to the boundary of the binarized calcaneus to ensure that only the bone structure was included in subsequent analyses.
3. Morphological hole filling: Internal cavities fully enclosed within the binarized bone structure were filled using morphological operations (see Figure 3B for illustration). This step ensured that enclosed voids did not artificially reduce the downstream surface area measurements.

*Bone volume (BV) and surface area (SA) calculations as conventional morphometric parameters****.*** Binary images obtained prior to hole filling were used to quantify BV, as these images preserve internal cavities relevant to volume measurements. In contrast, hole-filled binary images were used for calculation of SA, as filling internal voids provides a continuous bone surface necessary for accurate outer bone surface quantification. Following preprocessing, three-dimensional bone parameters were calculated from the final binary datasets (white and black voxel images) using Bruker analysis software. All measurements were performed using identical thresholding and processing parameters across samples to ensure consistency and comparability.

**Bone Roughness Analysis and Visualization Operator** (**BRAVO)-based analysis**

All steps (*Isosurface generation* and *Quantification of bone surface roughness*) were executed using BRAVO, our automated in-house MATLAB-workflow tailored for calcaneus-specific surface analysis.

*Isosurface generation.* After µCT acquisition, bone surface roughness was assessed using an isosurface-based framework designed to capture fine structural alterations, where an isosurface represents a surface extracted from volumetric data. The isosurface was computed from the same binary images after morphological hole filling (**μCT image processing and bone quantification**) used for SA calculation. To generate these surfaces, three-dimensional meshes were constructed using the marching cubes algorithm, a widely used geometric approach for volumetric surface reconstruction (4). Briefly, the algorithm evaluates individual voxels, detects surface intersections along voxel edges, and assembles triangular facets into a continuous mesh representation. Each surface mesh comprises a dense collection of triangular elements, with each triangle characterized by three vertices and an associated normal vector describing local surface orientation.

*Quantification of bone surface roughness.* Surface roughness associated with arthritic pathology was quantified using an approach based on surface normal vectors, adapted from previously described methodologies (5,6). For each triangular element within the isosurface mesh, angular differences were computed between its normal vector and those of neighboring triangles located within a circular vicinity. This procedure was iteratively applied across the full surface, yielding spatially resolved measurements of local surface irregularity.

Roughness information was visualized as surface maps to facilitate qualitative evaluation of erosion-related changes. For global quantification, roughness values were summarized using the integrated angle roughness (IAR) metric, following the established analytical framework (7,8). Angular deviation distributions were compiled into histograms and evaluated relative to composite histograms derived from healthy control samples to account for baseline surface variation. A roughness threshold (60°) was determined from the statistical properties of the control distribution, and angular deviations exceeding this threshold were classified as indicative of roughened surface regions (see Figure 3F for illustration). The IAR value was calculated by numerical integration of the frequency of the threshold angle across the analyzed surface.

**Statistical analysis**

Age-related effects were visualized using violin plots and quantified using receiver operating characteristic (ROC) area under the curve (AUC) analysis and Cohen’s d. ROC AUC was used as a threshold-independent measure of group discrimination (0.5 = random, 1.0 = perfect separation). Ordered logistic regression was performed using the statsmodels package (version 0.14.5). ROC/AUC analyses were conducted using scikit-learn (version 1.7.2), data handling using *pandas* (version 2.3.3) and *numpy* (version 2.3.3), and data visualization using *matplotlib* (version 3.10.6) and *seaborn* (version 0.13.2).

Principal component analysis (PCA) was performed using Orange Data Mining to visualize the relationship between the two input variables included in each analysis. For EMI analyses, PCA was performed using bone volume and surface area as input variables, whereas for BRAVO analyses, PCA was performed using bone surface irregularities (AUC T > 60°) and surface area units as input variables

**References**

1. Kouskoff V, Korganow A-S, Duchatelle V, et al. Organ-specific disease provoked by systemic autoimmunity. *Cell* 1996;87:811–822.

2. Monach PA, Mathis D, Benoist C. The k/bxn arthritis model. *Curr Protoc Immunol* 2008;Chapter 15:15.22.1-15.22.12.

3. Christensen AD, Haase C, Cook AD, et al. K/bxn serum-transfer arthritis as a model for human inflammatory arthritis. *Front Immunol* 2016;7:213.

4. Lorensen WE, Cline HE. Marching cubes: a high resolution 3d surface construction algorithm. *SIGGRAPH Comput Graph* 1987;21:163–169.

5. Silva MD, Savinainen A, Kapadia R, et al. Quantitative analysis of micro-ct imaging and histopathological signatures of experimental arthritis in rats. *Mol Imaging* 2004;3:15353500200404136.

6. Silva MD, Ruan J, Siebert E, et al. Application of surface roughness analysis on micro–computed tomographic images of bone erosion: examples using a rodent model of rheumatoid arthritis. *Mol Imaging* 2006;5:7290.2006.00025.

**Supplementary Figure 1. Clinical assessment of arthritis during the course of STIA**

Clinical score and ankle swelling in individual male C57BL/6J mice terminated at different time points during STIA. (n ≥ 7 per group; see Figure 1A). Each symbol represents an individual mouse. Bars represent means ± SEM. Statistical significance was determined by Kruskal-Wallis test followed by Dunn’s multiple comparisons test, with each time-point compared to Day 0. Only statistically significant comparisons are indicated in the figures. **P* < 0.05, ***P* < 0.01, *** *P* < 0.001, *****P* < 0.0001.

**Supplementary Figure 2. Semi-quantitative scoring of three-dimensional µCT scans reveals distinct phases of bone erosion and formation during STIA**

**A**) Overview of the workflow for μCT analysis of the hind paw. Acquired two-dimensional μCT images were reconstructed into a three-dimensional rendering, followed by slice alignment, segmentation of the calcaneus from the hind paw, parameter processing, and three-dimensional visualization using NRecon, DataViewer, and CTVox (Bruker software). Red boxes in the far-right images represents area used for scoring. **B**) Representative images of complete scoring scale used to assess bone erosion and bone formation in the hind paw and the corresponding calcaneus in the STIA model. Red arrows indicate areas of bone erosion and blue arrow indicate areas of bone formation. **C**,**D**) Bone erosion (**C**) and formation scores (**D**) of hind paw and corresponding calcaneus from mice shown in Figure 2B (n ≥ 7 per group; see Figure 1A). Each symbol in **C** and **D** represents an individual mouse. Bars represent mean ± SEM. Statistical significance in **C** and **D** was determined by Kruskal-Wallis test followed by Dunn’s multiple comparisons test, with each time-point compared to Day 0. Only statistically significant comparisons are indicated in the figures. **P* < 0.05, ***P* < 0.01, ****P* < 0.001, *****P* < 0.0001.

**Supplementary Figure 3. Quantitative µCT-based assessment of cortical bone remodeling during STIA using conventional morphometrics and the BRAVO-based workflow**

**A**) Representative µCT and mirrored heat mapped BRAVO images of C57BL/6J mice across all analyzed time-points, illustrating changes in bone surface. Red arrows indicating areas of bone erosion. Blue arrows indicating areas of bone formation. White arrows indicating areas with increased surface irregularity (roughness). **B**,**C**) EMI and BRAVO fold change across experimental time-point groups after STIA compared to day 0 (n ≥ 7 per group; see Figure 1A; Day 28 no STIA controls n = 6 [Day28 ctrl]). **D**) Receiver operating characteristic (ROC) analysis comparing BRAVO and EMI for detection of erosive lesions using blinded semi-quantitative µCT bone erosion scoring as the reference assessment. Samples with a semi-quantitative bone erosion score ≥1 were classified as positive. BRAVO demonstrated higher discriminatory performance than EMI for the detection of erosive lesions (AUC = 0.973, 95% CI 0.939–0.996 vs. AUC = 0.866, 95% CI 0.784–0.933). The difference between AUCs (ΔAUC = 0.108, 95% CI 0.040–0.187) was assessed using a paired bootstrap test and was statistically significant (*P* = 0.0006). Each symbol in **B** and **C** represents an individual mouse. Bars represent mean ± SEM. Statistical significance in **B** and **C** was determined by Kruskal-Wallis test followed by Dunn’s multiple comparisons test, with each time-point compared to Day 0. Only statistically significant comparisons are indicated in the figures. **P* <0.05, ***P* < 0.01, ****P* < 0.001, *****P* < 0.0001.

**Supplementary Figure 4. BRAVO provides robust and age-independent quantification of cortical bone remodeling during inflammatory arthritis**

**A**) Schematic overview of experimental set-up with mature adult (33 weeks) mice indicating number of mice analyzed at each time-point for downstream analysis. **B)** Clinical score and ankle swelling in C57BL/6J mice (n ≥ 5 per group; see **A**) subjected to STIA. **C**) Representative µCT and mirrored heat mapped BRAVO images of mature adult C57BL/6J mice across all time-point groups, illustrating changes in bone surface. Red arrows indicate areas of bone erosion. Blue arrows indicating areas of bone formation. White arrows indicate areas of increased surface irregularity (roughness). **D**) Surface area units (SAU) and Surface irregularity (SI) for mature adult mice (n ≥ 5 per group, see **A**) at different time-point groups after STIA. **E**) Scoring of bone erosion and bone formation on calcaneus across time-point groups after STIA (n ≥ 5 per group, see **A**). **F**,**G**) Violin plots with embedded box plots showing EMI and BRAVO distributions in non-arthritic (no STIA) young adult (12 [day 0] to 16 [day 28] weeks of age) (**F**) and mature adult (33 [day 0] to 37 [day 28] weeks of age) mice (n ≥ 11 per group) (**G**). For each comparison, group separability was quantified using the area under the ROC curve (AUC), defined as the probability that randomly selected sample is correctly ranked by the metric. Cohen’s d was calculated as the standardized mean difference, defined as the difference between group means divided by the pooled standard deviation, describing how distinct the two groups are. Each symbol in **B,D** and **E** represents an individual mouse. Bars represent mean ± SEM. Statistical significance in **D,E** was determined by Kruskal-Wallis test followed by Dunn’s multiple comparisons test, with each time-point compared to Day 0. Only statistically significant comparisons are indicated in the figures. *P < 0.05, **P < 0.01, ***P < 0.001.

**Supplementary Figure 5. Kinetic joint transcriptional profiling of inflammatory, bone-remodeling and extracellular matrix remodeling genes during STIA**

**A**-**C)** Expression of inflammation-related genes, bone remodeling genes and extracellular matrix remodeling genes in hind paws from mice (n ≥ 7 per group; see Figure 1A) at different time points during STIA. Relative gene expression was calculated using the ΔCt method (gene of interest - *Gapdh*). Graphs represent mean ± SEM. Statistical significance in **A** to **C** was determined by Kruskal-Wallis test followed by Dunn’s multiple comparisons test, with each time-point compared to Day 0. Only statistically significant comparisons are indicated in the figures. **P* < 0.05, ***P* < 0.01, ****P* < 0.001, *****P* < 0.0001.

**Supplementary Figure 6. Treatment-induced suppression of cortical bone erosion during STIA assessed by conventional morphometric and BRAVO-derived parameters**

**A**,**B**) Clinical score of C57BL/6J mice treated with either an anti-RANKL (αRANKL) antibody or an IgG isotype control during STIA. Mice were terminated at either day 14 (**A**) or day 28 (**B**) after STIA induction. Treatment was initiated on day 3 after STIA induction. Mice terminated on day 14 received treatment on days 3, 5, 7, 10 and 12. Mice terminated on day 28 received treatment on day 3, 5, 7, 10, 12 and 14. Arrows indicate the time points of intraperitoneal (i.p) antibody injection (n ≥ 9 per group; See Figure 6A). Differences in arthritis severity were quantified using the area under the curve (AUC). **C**,**D**) Semi-quantitative scoring of bone erosion and bone formation in the hind paw between treatment groups at day 14 and day 28 after STIA (n ≥ 9 per group, see Figure 6A). **E**) Quantitative measurements of calcaneus Bone Volume (BV) and Surface Area (SA) between treatment groups at day 14 and day 28 after STIA (n ≥ 9 per group, see Figure 6A). **F**) Surface Area Units (SAU) and Surface Irregularity (SI) between treatment groups at day 14 and day 28 after STIA (n ≥ 9 per group). Graphs represent mean ± SEM. Statistical significance in **A**-**F** was determined using the Mann-Whitney U test. Only statistically significant comparisons are indicated in the figures. **P* < 0.05, ***P* < 0.01, ****P* < 0.001, *****P* < 0.0001.

**Supplementary table 1. Primer sequences (mouse)**

*Below qPCR primers were purchased from Sigma-Aldrich (Merck).*

| **Primer names** | **Primer sequences (5’-3’) Forward + Reverse (F + R)** |
| --- | --- |
| *Ctsk* | F: CAGAGGGTACAGAGAGATTC  R: TCATCATAGTACACACCTCTG |
| *Alpl* | F: GGATCTCATCAGTAATTTGGAAG  R: AAAGAGACCTAAGAGGTAGTC |
| *Tnfsf11* | F: TCTGTTCCTGTACTTTCGAG  R: TTCATGGAGTCTCAGGATTC |
| *Bglap* | F: ACCATGAGGACCATCTTTC  R: GGACATGAAGGCTTTGTC |
| *Tnfrs11b* | F: GAAGATCATCCAAGACATTGAC  R: TCCTCCATAAACTGAGTAGC |
| *Runx2* | F: ACAAGGACAGAGTCAGATTAC  R: CAGTGTCATCATCTGAAATACG |
| *Nfatc1* | F: CCAAGTCAGTTTCTATGTCTG  R: ATAATTGGAACATTGGCAGG |
| *Sp7* | F: TGCTTGAGGAAGAAGCTC  R: CTTCTTTGTGCCTCCTTTC |
| *Mmp2* | F: GAGATCTTCTTCTTCAAGGAC  R: AATAGACCCAGTACTCATTCC |
| *Mmp3* | F: CAAATACTGGAGGTTTGATGAG  R: CTCTTCAATATGTGGGTCAC |
| *Mmp7* | F: CAAGGAGAGATCATGGAGAC  R: CATCAGTCCAGTACTCATCC |
| *Mmp9* | F: CTTCCAGTACCAAGACAAAG  R: ACCTTGTTCACCTCATTTTG |
| *Mmp10* | F: CACCGTGAAGAAGATTGATG  R: CAAATCTCCAGTATTTGTCCC |
| *Mmp12* | F: AACACTACTGGAGGTATGATG  R: CTGCATCAATTTTAGGCTTG |
| *Mmp13* | F: CTTTAGAGGGAGAAAATTCTGG  R: CATCATCATAACTCCACACG |
| *Mmp14* | F: CTTCAAAGGAGATAAGCACTG  R: AGAAGTAGGTCTTCCCATTG |
| *Col1a1* | F: CGTATCACCAAACTCAGAAG  R: GAAGCAAAGTTTCCTCCAAG |
| *Col1a2* | F: CAGCGATTACTACTGGATTG  R: GATAGTCTCTCCTAACCAGAC |
| *Tnf* | F: CTATGTCTCAGCCTCTTCTC  R: CATTTGGGAACTTCTCATCC |
| *Il1β* | F: GGATGATGATGATAACCTGC  R: CATGGAGAATATCACTTGTTGG |
| *Csf1* | F: TAGAAAGGATTCTATGCTGGG  R:CTCTTTGGTTGAGAGTCTAAG |
| *Csf2* | F: CTACTACCAGACATACTGCC  R: GCATTCAAAGGGGATATCAG |
| *Cxcl10* | F: AAAAAGGTCTAAAAGGGCTC  R: AATTAGGACTAGCCATCCAC |
| *Ccl5* | F: AGGAGTATTTCTACACCAGC  R: CAGGGTCAGAATCAAGAAAC |
| *Nos2* | F: CATCAACCAGTATTATGGCTC  R: TTTCCTTTGTTACAGCTTCC |
| *Ccl3* | F: TTCTCTGTACCATGACACTC  R: CTCTTAGTCAGGAAAATGACAC |
| *Cxcl1* | F: AAAGATGCTAAAAGGTGTCC  R: GTATAGTGTTGTCAGAAGCC |
| *Cxcl2* | F: GGGTTGACTTCAAGAACATC  R: CCTTGCCTTTGTTCAGTATC |
| *Cxcl5* | F: TGTTTGCTTAACCGTAACTC  R: CAGTTTAGCTATGACTTCCAC |
| *Cd200* | F: GTCTGGACAAAGGATTTTGG  R: GATGGAGATCAAGATCAGAAG |
| *Dkk1* | F: ATATCACACCAAAGGACAAG  R: CCTTCTTTAAGGACAGGTTTAC |
| *Sost* | F: TCAGGAATGATGCCACAG  R: GTACTCGGACACATCTTTG |
| *Tgfb1* | F: GGATACCAACTATTGCTTCAG  R: TGTCCAGGCTCCAAATATAG |

Primers for *Il6* and *Gapdh* were obtained from Qiagen. Primer sequences are proprietary and available from the manufacturer upon request.

**Supplementary Table 2A. Inflammatory gene expression over time**

| Gene | Day 0 | Day 3 | Day 7 | Day 10 | Day 14 | Day 17 | Day 21 | Day 24 | Day 28 |
| --- | --- | --- | --- | --- | --- | --- | --- | --- | --- |
| *Ccl3* | 0.00024 (0.00088) n=10 | 0.00059 (0.00263) n=11 | 0.00241 (0.00749) n=9 | 0.00534 (0.00914) n=9 | 0.00258 (0.00859) n=9 | 0.00147 (0.00468) n=10 | 0.00043 (0.00088) n=10 | 0.00061 (0.00100) n=7 | 0.00028 (0.00137) n=10 |
| *Ccl5* | 0.00050 (0.00197) n=11 | 0.00074 (0.00649) n=11 | 0.02549 (0.04658) n=9 | 0.03491 (0.12127) n=9 | 0.01721 (0.10284) n=9 | 0.00864 (0.09577) n=10 | 0.00147 (0.01680) n=10 | 0.00111 (0.06492) n=7 | 0.00160 (0.01367) n=10 |
| *Csf1* | 0.00179 (0.00348) n=11 | 0.00207 (0.00593) n=11 | 0.00784 (0.01185) n=9 | 0.00553 (0.01358) n=9 | 0.00702 (0.00992) n=9 | 0.00364 (0.00867) n=10 | 0.00279 (0.00648) n=10 | 0.00354 (0.04120) n=7 | 0.00194 (0.00682) n=10 |
| *Csf2* | 0.00005 (0.00037) n=11 | 0.00004 (0.00022) n=10 | 0.00035 (0.00063) n=9 | 0.00033 (0.00174) n=9 | 0.00042 (0.00351) n=9 | 0.00029 (0.00155) n=8 | 0.00017 (0.00173) n=8 | 0.00012 (0.00091) n=6 | 0.00012 (0.00061) n=9 |
| *Cxc1* | 0.00100 (0.00832) n=11 | 0.01009 (0.08495) n=11 | 0.22300 (0.34570) n=9 | 0.19570 (0.51507) n=9 | 0.07124 (0.25314) n=9 | 0.00822 (0.28022) n=10 | 0.00214 (0.00526) n=10 | 0.00231 (0.02439) n=7 | 0.00320 (0.01910) n=10 |
| *Cxcl2* | 0.00007 (0.00163) n=11 | 0.00115 (0.01018) n=11 | 0.01347 (0.06655) n=9 | 0.02274 (0.03291) n=9 | 0.01135 (0.05209) n=9 | 0.00106 (0.03606) n=10 | 0.00036 (0.00068) n=10 | 0.00013 (0.00083) n=7 | 0.00020 (0.00330) n=10 |
| *Cxcl5* | 0.00007 (0.03043) n=11 | 0.00155 (0.04293) n=11 | 0.46810 (2.03360) n=9 | 0.53990 (1.21085) n=9 | 0.05529 (0.56539) n=9 | 0.00126 (0.43770) n=10 | 0.00014 (0.00134) n=10 | 0.00035 (0.01113) n=7 | 0.00010 (0.00543) n=10 |
| *Cxcl10* | 0.00102 (0.00387) n=11 | 0.00292 (0.01701) n=11 | 0.06178 (0.10289) n=9 | 0.06746 (0.12521) n=9 | 0.01612 (0.14495) n=9 | 0.00853 (0.07154) n=10 | 0.00188 (0.00609) n=10 | 0.00513 (0.02724) n=7 | 0.00189 (0.01783) n=10 |
| *Il1β* | 0.00009 (0.00070) n=11 | 0.00526 (0.01955) n=11 | 0.05823 (0.24696) n=9 | 0.07935 (0.27394) n=9 | 0.01764 (0.17185) n=9 | 0.00129 (0.19460) n=10 | 0.00029 (0.00296) n=10 | 0.00054 (0.07891) n=7 | 0.00032 (0.00194) n=10 |
| *Il6* | 0.00049 (0.00104) n=11 | 0.00816 (0.01831) n=11 | 0.07091 (0.27283) n=9 | 0.07626 (0.26249) n=9 | 0.00920 (0.26122) n=9 | 0.00224 (0.19592) n=9 | 0.00083 (0.00871) n=10 | 0.00082 (0.00979) n=6 | 0.00068 (0.01000) n=10 |
| *Nos2* | 0.00007 (0.00019) n=11 | 0.00018 (0.00055) n=11 | 0.00195 (0.00261) n=9 | 0.00195 (0.00198) n=9 | 0.00167 (0.00468) n=9 | 0.00045 (0.00420) n=10 | 0.00021 (0.00053) n=10 | 0.00018 (0.00098) n=7 | 0.00017 (0.00065) n=10 |
| *Tnf* | 0.00010 (0.00035) n=11 | 0.00018 (0.00057) n=11 | 0.00159 (0.00354) n=9 | 0.00126 (0.00184) n=9 | 0.00069 (0.00352) n=9 | 0.00042 (0.00215) n=10 | 0.00028 (0.00061) n=10 | 0.00016 (0.00030) n=6 | 0.00013 (0.00055) n=10 |
| *Cd200* | 0.00659  (0.05343)  n=11 | 0.00597  (0.04945)  n=11 | 0.04803  (0.03345)  n=9 | 0.03051  (0.05981)  n=10 | 0.00942  (0.04396)  n=9 | 0.02691  (0.08288)  n=10 | 0.01021  (0.03344)  n=10 | 0.00258  (0.00846)  n=7 | 0.01497  (0.02331)  n=10 |

Data are presented as median (interquartile range). n = number of mice analyzed at defined time-point**.**

**Supplementary Table 2B. Osteoclast and osteoblast marker expression over time**

| Gene | Day 0 | Day 3 | Day 7 | Day 10 | Day 14 | Day 17 | Day 21 | Day 24 | Day 28 |
| --- | --- | --- | --- | --- | --- | --- | --- | --- | --- |
| *Alpl* | 0.00158 (0.00423) n=11 | 0.00302 (0.00500) n=11 | 0.02894 (0.03228) n=9 | 0.05741 (0.08215) n=9 | 0.02890 (0.11436) n=9 | 0.03792 (0.22248) n=10 | 0.01208 (0.10357) n=10 | 0.01570 (0.19418) n=7 | 0.01490 (0.03832) n=10 |
| *Bglap* | 0.01784 (0.17465) n=11 | 0.02498 (0.07914) n=11 | 0.04046 (0.24869) n=9 | 0.27240 (0.44120) n=9 | 0.33560 (2.50171) n=9 | 0.88520 (9.61820) n=10 | 0.22150 (7.76909) n=10 | 0.31370 (2.00154) n=7 | 0.26870 (1.58021) n=10 |
| *Ctsk* | 0.05910 (0.12121) n=11 | 0.05862 (0.23137) n=11 | 3.34100 (5.65600) n=9 | 2.44000 (3.49200) n=9 | 1.68400 (4.15250) n=9 | 2.25500 (8.37980) n=10 | 0.56500 (5.80781) n=9 | 0.49900 (3.68772) n=6 | 0.75160 (1.66124) n=10 |
| *Nfatc1* | 0.00190 (0.00297) n=11 | 0.00174 (0.00202) n=11 | 0.01087 (0.01093) n=9 | 0.01093 (0.00565) n=9 | 0.01120 (0.01210) n=9 | 0.00709 (0.01246) n=10 | 0.00303 (0.00728) n=10 | 0.00421 (0.00674) n=7 | 0.00377 (0.00562) n=10 |
| *Runx2* | 0.00018 (0.00159) n=11 | 0.00047 (0.00084) n=11 | 0.00600 (0.01011) n=9 | 0.00911 (0.01347) n=9 | 0.00554 (0.02375) n=9 | 0.00605 (0.03838) n=10 | 0.00221 (0.01005) n=10 | 0.00352 (0.00530) n=7 | 0.00151 (0.01207) n=10 |
| *Sp7* | 0.00014 (0.00045) n=11 | 0.00023 (0.00099) n=11 | 0.00509 (0.00813) n=9 | 0.01125 (0.01505) n=9 | 0.00315 (0.01545) n=9 | 0.00460 (0.01555) n=10 | 0.00128 (0.00777) n=10 | 0.00194 (0.00517) n=7 | 0.00164 (0.00402) n=10 |
| *Tnfrsf11b* | 0.00404 (0.00937) n=11 | 0.00397 (0.00891) n=11 | 0.01300 (0.03405) n=9 | 0.01884 (0.03429) n=9 | 0.01055 (0.04032) n=9 | 0.01372 (0.03433) n=10 | 0.00816 (0.02885) n=10 | 0.00783 (0.02419) n=7 | 0.01089 (0.04729) n=10 |
| *Tnfsf11* | 0.00008 (0.00026) n=11 | 0.00114 (0.00418) n=10 | 0.03275 (0.09675) n=9 | 0.04138 (0.05279) n=9 | 0.01005 (0.07824) n=9 | 0.00541 (0.08232) n=10 | 0.00154 (0.00850) n=10 | 0.00072 (0.00590) n=7 | 0.00178 (0.00514) n=10 |
| *Dkk1* | 0.00031  (0.00417)  n=11 | 0.00019  (0.00237)  n=11 | 0.00333  (0.00574)  n=9 | 0.00137  (0.00819)  n=10 | 0.00031  (0.00211)  n=9 | 0.00128  (0.00544)  n=10 | 0.00044  (0.00135)  n=10 | 0.00014  (0.00085)  n=7 | 0.00053  (0.00173)  n=10 |
| *Sost* | 0.00055  (0.00060)  n=11 | 0.00051  (0.00261)  n=11 | 0.00075  (0.00121)  n=9 | 0.00075  (0.00199)  n=10 | 0.00052  (0.00237)  n=9 | 0.00080  (0.00240)  n=10 | 0.00044  (0.00166)  n=10 | 0.00051  (0.00058)  n=6 | 0.00066  (0.00137)  n=10 |

Data are presented as median (interquartile range). N = number of mice analyzed at defined time-point.

**Supplementary Table 2C. MMPs and Collagen type I expression over time**

| Gene | Day 0 | Day 3 | Day 7 | Day 10 | Day 14 | Day 17 | Day 21 | Day 24 | Day 28 |
| --- | --- | --- | --- | --- | --- | --- | --- | --- | --- |
| Col1a1 | 0.02066 (0.07916) n=11 | 0.03124 (0.09175) n=11 | 1.10000 (4.03910) n=9 | 1.54000 (2.46490) n=9 | 0.49670 (2.75180) n=9 | 0.66770 (2.31819) n=10 | 0.37140 (0.86178) n=10 | 0.08385 (0.58063) n=7 | 0.14390 (0.63916) n=10 |
| Col1a2 | 0.96690 (4.94280) n=11 | 1.00100 (1.61350) n=11 | 19.46000 (72.03000) n=8 | 53.03000 (222.25000) n=9 | 32.19000 (183.50600) n=8 | 28.38000 (213.15600) n=10 | 13.67000 (123.76300) n=10 | 14.23000 (50.93240) n=7 | 14.33000 (60.78700) n=10 |
| Mmp2 | 0.02981 (0.04580) n=11 | 0.04910 (0.07710) n=11 | 0.58730 (0.97670) n=9 | 1.32200 (1.63800) n=9 | 1.30500 (2.31870) n=9 | 0.94130 (2.39358) n=10 | 0.35340 (1.17783) n=10 | 0.51080 (0.90045) n=6 | 0.41140 (1.05584) n=10 |
| Mmp3 | 0.00450 (0.01658) n=11 | 0.06455 (0.90675) n=11 | 2.35200 (8.20836) n=9 | 3.13100 (6.20470) n=9 | 1.38600 (21.13477) n=9 | 0.82450 (14.94970) n=10 | 0.12500 (1.70468) n=10 | 0.14080 (0.96335) n=7 | 0.11190 (1.83552) n=10 |
| Mmp7 | 0.000005 (0.00010) n=11 | 0.00003 (0.00008) n=10 | 0.00083 (0.00185) n=9 | 0.00146 (0.00443) n=9 | 0.00033 (0.00351) n=9 | 0.00015 (0.00072) n=9 | 0.00003 (0.00018) n=9 | 0.00012 (0.00044) n=7 | 0.00002 (0.00008) n=9 |
| Mmp9 | 0.00046 (0.00366) n=11 | 0.00181 (0.03851) n=11 | 0.72910 (0.96020) n=9 | 0.38290 (0.58120) n=9 | 0.30520 (1.26443) n=9 | 0.17870 (1.63588) n=10 | 0.03900 (0.50373) n=10 | 0.08090 (0.36565) n=7 | 0.04042 (0.28372) n=10 |
| Mmp10 | 0.00035 (0.02317) n=11 | 0.00018 (0.00233) n=11 | 0.00700 (0.01072) n=9 | 0.00725 (0.01485) n=9 | 0.01173 (0.01693) n=9 | 0.01299 (0.02766) n=10 | 0.00126 (0.00530) n=10 | 0.00118 (0.00890) n=7 | 0.00089 (0.00269) n=10 |
| Mmp12 | 0.00323 (0.04414) n=11 | 0.00189 (0.01355) n=11 | 0.01041 (0.02273) n=9 | 0.02123 (0.0845) n=9 | 0.01311 (0.1711) n=9 | 0.02051 (0.2062) n=10 | 0.00399 (0.02944) n=10 | 0.00293 (0.04568) n=7 | 0.00398 (0.03211) n=10 |
| Mmp13 | 0.00089 (0.00378) n=11 | 0.00550 (0.09711) n=11 | 0.90260 (1.89090) n=9 | 1.44000 (2.08300) n=9 | 1.34500 (6.31090) n=9 | 1.13100 (5.09479) n=10 | 0.17790 (0.95845) n=10 | 0.14630 (1.60684) n=7 | 0.18370 (0.58498) n=9 |
| Mmp14 | 0.00723 (0.00939) n=11 | 0.01220 (0.03381) n=11 | 0.33530 (0.77043) n=9 | 0.48810 (0.48210) n=9 | 0.38300 (1.06370) n=9 | 0.35350 (0.76762) n=10 | 0.07771 (0.30883) n=10 | 0.07391 (0.34603) n=7 | 0.05801 (0.16145) n=10 |
| *Tgfb1* | 0.04583  (0.26876)  n=11 | 0.03946  (0.33715)  n=11 | 0.17740  (0.21470)  n=9 | 0.17190  (0.31416)  n=10 | 0.05857  (0.10823)  n=9 | 0.09999  (0.22487)  n=10 | 0.06595  (0.13318)  n=10 | 0.02578  (0.05033)  n=7 | 0.08974  (0.23923)  n=10 |

Data are presented as median (interquartile range). n = number of mice analyzed at defined time-point.

Page left intentionally blank.
